# The only complete articulated early Miocene chameleon skull (Rusinga Island, Kenya) suggests an African origin for Madagascar’s endemic chameleons

**DOI:** 10.1038/s41598-019-57014-5

**Published:** 2020-01-10

**Authors:** Andrej Čerňanský, Anthony Herrel, Job M. Kibii, Christopher V. Anderson, Renaud Boistel, Thomas Lehmann

**Affiliations:** 1 Department of Ecology, Laboratory of Evolutionary Biology, Faculty of Natural Sciences, Comenius University in Bratislava, 84215 Bratislava, Mlynská dolina Slovakia; 2 Département Adaptation du Vivant, UMR 7179C.N.R.S/ Muséum National d’Histoire Naturelle, 55 rue Buffon, 75005 Paris, France; 3 Earth Sciences Department, National Museums of Kenya, Nairobi, Kipande Road P.O. BOX, 40658– 00100 Nairobi, Kenya; 4 University of South Dakota, Department of Biology, 414 E. Clark Street – UCL 191, Vermillion South Dakota, USA; 5 Muséum National d’Histoire Naturelle, UMR 7179C.N.R.S/M.N.H.N., Bâtiment d’Anatomie Comparée, 55 rue Buffon, CP 55, 75005 Paris, France; 6 Senckenberg Research Institute and Natural History Museum Frankfurt, Department of Messel Research and Mammalogy, Senckenberganlage 25, 60325 Frankfurt, am Main Germany

**Keywords:** Ecology, Evolution, Zoology

## Abstract

We here present the first detailed study of the specimen KNM-RU 18340 from Rusinga Island (Kenya), the only known complete early Miocene chameleon skull, using micro-CT. This specimen represents one of the oldest chameleon fossils ever recovered. For the first time, the skull bone internal surfaces, their sutures, and elements contained inside the rocky matrix are observed. Our morphological comparisons and phylogenetic analyses place this specimen confidently in the genus *Calumma* and a new species, *Calumma benovskyi* sp. nov., is erected for it. Since all species of this genus are endemic to Madagascar, this fossil uniquely demonstrates the existence of *Calumma* on continental Africa in the past. Our results challenge the long-held view that chameleons originated on Madagascar and dispersed over water to Africa, and provide a strong evidence of an African origin for some Malagasy lineages. The Oligocene–early Miocene dispersal to Madagascar, using oceanic currents that favoured eastward dispersal at that time, is a highly supported scenario matching the suggested dispersal of lemurs to this island. This is consistent with a previously suggested hypothesis based on molecular data.

## Introduction

The Chamaeleonidae is an unusual family of lizards including extant species from Africa, Madagascar, the Middle East, southern India, Sri Lanka, and the Mediterranean region of Europe. It is a highly characteristic and morphologically specialized clade of acrodont iguanians. This clade is mainly composed of arboreal forms, but includes a ground-dwelling desert form –the Namaqua chameleon^1,2^. About half of the accepted species of chameleons occur in Madagascar. This island has therefore been suggested to be a centre of diversity of the clade from where it likely radiated via oceanic dispersal^3^. Later, in contrast, the molecular phylogeny of Tolley *et al*.^4^ suggested that the family most probably originated in Africa, with two separate oceanic dispersal events to Madagascar during the Palaeocene and the Oligocene, when prevailing oceanic currents would have favoured eastward dispersal. The fossil record of these animals, the only form of direct evidence regarding the early evolution and palaeobiogeography of these animals, is unfortunately scant. Thus, a key element for resolving this conundrum is lacking.

Molecular data suggest a Cretaceous origin^4^, but the oldest known fossil record of crown members only dates back to the early Miocene (MN3)^5,6^. Chamaeleonid fossils are mostly reported from the Miocene of Europe^6–12^ and are also known from Africa^13,14^ and potentially India^15^. Further African fossils have been documented from early Pliocene deposits^16,17^. The vast majority of the fossil record is represented by isolated elements, mostly jaw fragments, which renders taxonomical identification to the genus or species level often difficult or impossible. Only two notable exceptions exist - *Chamaeleo intermedius*, based on a natural calcite cast described by Hillenius^13^ from the middle Miocene of Fort Ternan in Kenya; and *Ch. andrusovi* from the early (and potentially middle) Miocene of Europe, based on well-documented isolated cranial elements described by Čerňanský^6^.

**Figure 1:**
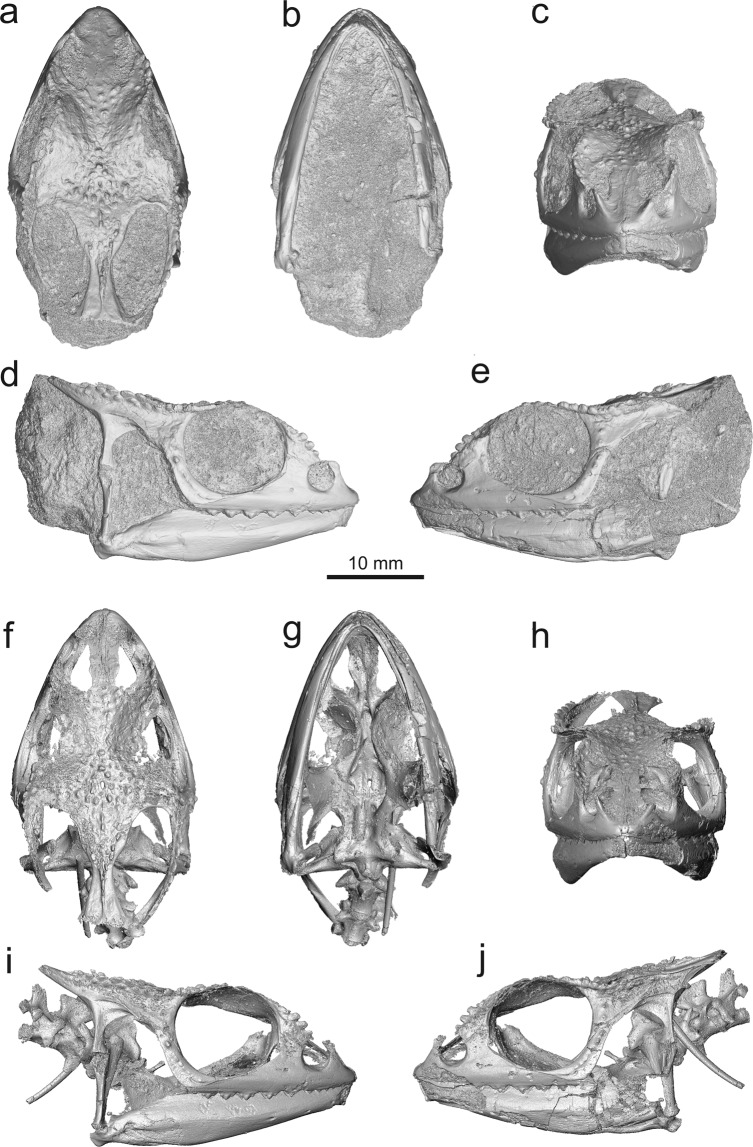
*Calumma benovskyi* sp. nov., the holotype KNM-RU 18340 from the lower Miocene of Kenya in (**a**,**f**) dorsal; (**b**,**g**) ventral; (**c**,**h**) anterior; (**d**,**i**) right; and (**e**,**j**) left views. These 3D models of the specimen were obtained by µCT and show the specimen in its original preservation condition (with a sedimentary matrix) on the upper side (**a**–**e**), whereas the lower side (**f**–**j**) shows the specimen with the sediment virtually removed by digital means.

The fossil locality of Rusinga Island (early Miocene, Lake Victoria, Kenya) is famous for its numerous fossil mammals^18^ (for geology see Supplementary Data 1), but this locality also yielded numerous reptile specimens such as varanids, crocodiles^19,20^, as well as the almost complete, and remarkably three-dimensionally preserved, skull of a fossil chamaeleonid (KNM-RU 18340; National Museums of Kenya). The skull is not fully freed from the rock, as matrix fills the entire internal region and covers many bone elements (Fig. 1). The specimen has been only preliminary reported by Rieppel *et al*.^14^. These authors suggested that it may be an early representative of the genus *Rhampholeon*, but their report was based on a cast of the specimen and photographs of the original only. Importantly, this specimen represents the only known complete fossil chameleon skull from the early Miocene. Thus, detailed knowledge on the morphology of this fossil African chameleon and its taxonomy is crucial to our understanding of the evolution of this lizard clade and for understanding African paleobiogeography and possible dispersal events.

The aims of this paper are: 1) to use high-resolution X-ray microcomputed tomography (μCT) to virtually prepare all skeletal elements of KNM-RU 18340; 2) to discover potentially hidden bones embedded in the sediment not visible from the outside; and 3) on the basis the obtained new anatomical data to evaluate the possible taxonomic position of KNM-RU 18340 and shed light on scenarios for the origin and diversification of chameleons.

## Systematic Palaeontology

Squamata Oppel, 1811^21^

Chamaeleonidae Gray, 1825^22^

*Calumma* Gray, 1865^23^

*Calumma benovskyi* sp. nov.

### Etymology

After Count Móric Beňovský (also spelled Benyovszky). He was born in Slovakia (Vrbové, 9. 20. 1746) and died at the age of 39 in Madagascar (5. 23. 1786). He is known as an important traveler and adventurer - the first European to sail in the North Pacific Ocean (seven years before James Cook) and the first person who explored Saint Lawrence Island. He traveled to Madagascar, where local tribal chiefs elected him as a “king” (ampansacabe) of Madagascar in 1776. His story parallels the history of *Calumma* - born abroad, he reached the island by sailing across the ocean.

### Holotype

KNM-RU 18340 (field number RU 1916'87; National Museums of Kenya, Nairobi): skull, mandible and three cervical vertebrae in connection (Figs. 1, 2 and Supplementary Figs. 1–13).

**Figure 2:**
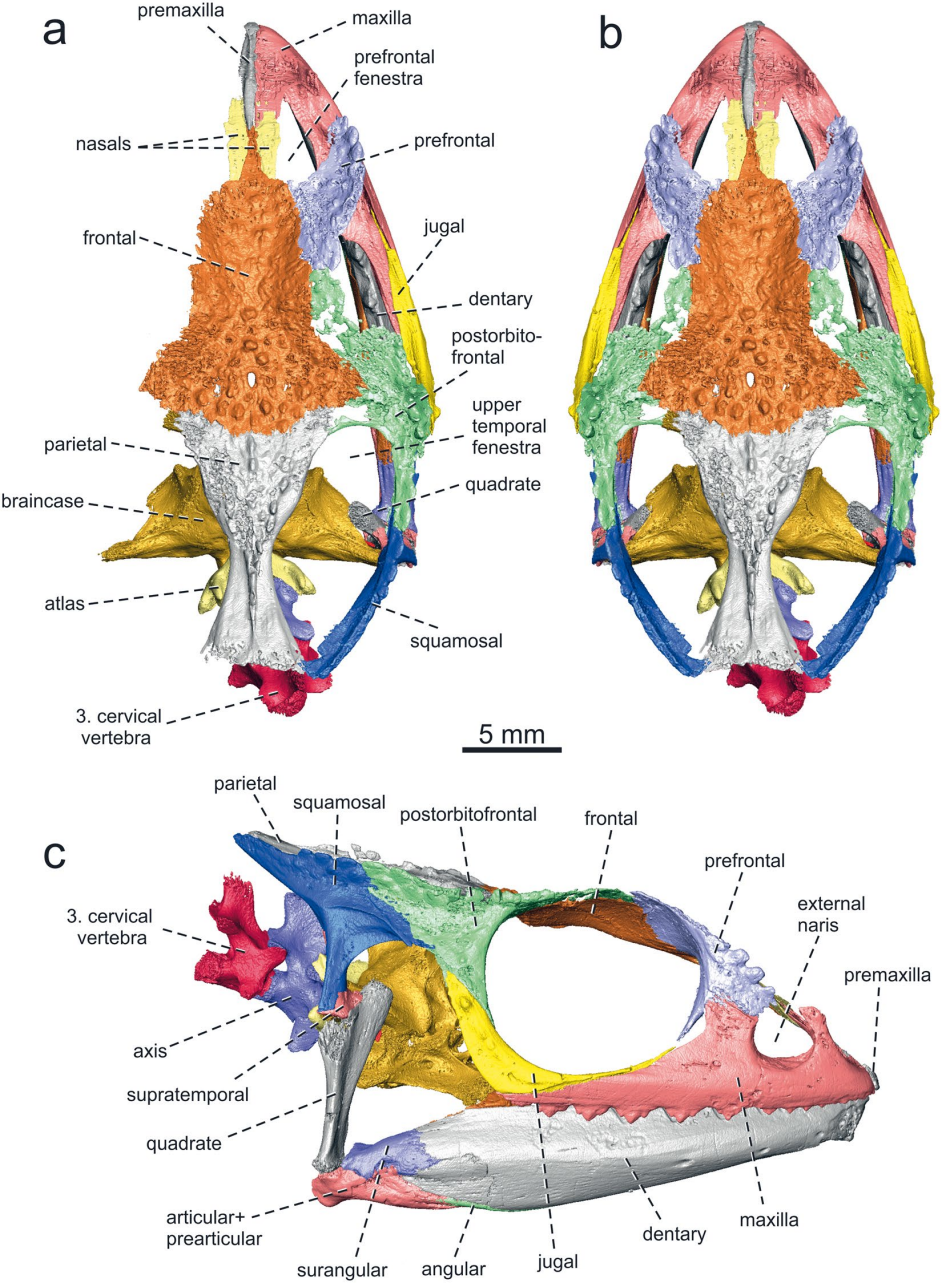
*Calumma benovskyi* sp. nov., the holotype KNM-RU 18340 from the lower Miocene of Kenya with virtually segmented bones from the dorsal and right side (best preserved elements) in (**a**) dorsal view; (**b**) the reconstruction of the complete specimen in dorsal view restored by mirror imaging; and (**c**) virtually segmented bones in right lateral view.

### Type locality

Rusinga Island (Lake Victoria, Kenya, Africa), site R107.

### Age

Early Miocene, base of the Hiwegi Formation.

### Diagnosis

A species of *Calumma* distinguishable from other species by the posterior corners of the frontal, forming well-developed posterolateral processes. Besides this feature, this taxon is characterized by the unique combination of the following characters: (1) nasal-frontal contact present; (2) prefrontal fenestra present and connected to external naris; (3) prefrontal inclined laterally in dorsal view; (4) contact between prefrontal and postorbitofrontal, excluding the frontal from the orbit; (5) orbital margin medially concave rather than straight in dorsal view; (6) single line of protuberances on the external surface of jugal; (7) hour-glass shaped parietal, bearing sculpture arrangement roughly resembling the Greek letter Ψ (psi); (8) well-developed and rather regularly distributed sculpture formed by protuberances on the dorsal surface of the frontal; (9) frontal completely pierced by the parapineal foramen; (10) posterodorsal process of postorbitofrontal reaching above the orbit, inducing an horizontal upper temporal fenestra, dorsally open, and not visible in lateral view; (11) posterodorsal process of squamosal more or less horizontal rather than vertical; and (12) the maximum width of the skull in dorsal view located at the level of the frontal-parietal contact rather than posterior to it.

#### General description and comparison

In dorsal view, the skull presents an ovoid shape, with its widest dimension being at the level of the frontal-parietal contact (Fig. 2A,B). In lateral aspect, the skull is triangular and gradually tapers anteriorly (Fig. 2C). The orbits are large, their anteroposterior length forms 1/3 of the complete anteroposterior length of the skull. The casque is flat and weakly elevated posteriorly, without a dorsally convex parietal. For detailed description and ilustration of each virtually isolated element, see Supplementary Data 1. The specimen KNM-RU 18340 shares the following features mainly with members of extant *Calumma* (see also Supplementary Figs. 14–16):

1. The contact of the premaxilla with the frontal. This is present in *Calumma globifer*, *Ca. boettgeri* and *Ca. gubei*, but not in *Ca. parsonii*, *Ca. brevicorne*, *Ca. nasutum*, *Ca. fallax*, and *Ca. juliae*. It is present in *Furcifer pardalis* and occasionally in *F. oustaleti* (but not in *F. bifidus* and *F. campani*)^24,25^. Is is also present in *Rhampholeon* and *Rieppeleon*, but not in *Bradypodion*, *Chamaeleo*, *Kinyongia*, *Trioceros* and *Brookesia*. The premaxilla-frontal contact is absent in the outgroup and character optimization in Mesquite supports this character state as being the condition at the basal node of the Chamaeleonidae clade. Character optimization in Mesquite shows that the contact of these two bones evolved independently in *Rieppeleon*, *Rhampholeon* and some members of *Calumma*. Among *Calumma*, the absence of the contact is at the basal node of the clade. So its presence can be regarded as derived among of this clade.
2. The paired nasals do not contact one another and are separated by a strut of bone formed by the premaxilla and the frontal. The majority of the chameleons have paired nasals, whereas *Bradypodion* and *Brookesia* often have a single nasal^24^.
3. The prefrontal fenestra is connected to the external naris (contra Rieppel *et al*.^14^). This character state can be observed in some extant chameleons, such as *Calumma nasutum*, *Ca. fallax*, *Ca. vatosoa*, *Ca. guibei* (but not in *Ca. globifer*, *Ca. parsonii*, *Ca. brevicorne, Ca. boettgeri* and *Ca. gehringi*), but also in *Rhampholeon*, *Archaius* and *Trioceros melleri*^24–26^. This connection is absent in e.g., *Brookesia*, *Rieppeleon*, *Nadzikambia*, *Bradypodion*, *Chamaeleo* and *Furcifer*. As for *Calumma*, character optimization in Mesquite supports absence of the connection as being the condition at the basal node of the clade, whereas its presence is derived.
4. The single line of protuberances on the postorbital process of the jugal. Two lines are present e.g., *Calumma ambreense*, *Ca. parsonii* and in the European early Miocene *Chamaeleo andrusovi*^6^, whereas one line is present in *Ca. brevicorne*.
5. The suborbital process of the jugal in lateral aspect; only a dorsal narrow portion of this process is exposed dorsal to maxilla. This is present in *Calumma*, *Chamaeleo*, *Trioceros*, *Kinyongia*, *Rhampholeon* and *Brookesia*. It is largerly exposed in *Rieppeleon* and *Bradypodion*, where, moreover, the process often bears a sculpture formed by protuberances. It is also largerly exposed in *Uromastyx* (outgroup). However, the suborbital process in lateral view is covered by the maxilla in *Archaius* and many *Furcifer* species (not in, e.g, *F. lateralis*, where a narrow portion is exposed)^24^.
6. The absence of jugal - squamosal contact. This contact is absent in *Calumma* (except for *Ca. parsonii*), *Brookesia*, *Rieppeleon* and *Bradypodion*, whereas the contact is usually present in members of the genera *Chamaeleo*, *Nadzikambia*, *Archaius*, *Kinyongia*, and some members of *Furcifer* (not in e.g., *F. campani*) and *Trioceros*^24^. The character optimization in Mesquite supports the absence of jugal - squamosal contact as being the condition at the basal node of the clade formed by all chameleons. Character optimization in Mesquite shows that presence of this contact is the condition at the basal node of the clade [*Chamaeleo* + *Trioceros* + *Kinyongia* + *Furcifer* + *Calumma*], whereas the contact of these bones in *Archaius* evolved independently. The absence of this contact in members of *Calumma* is regarded as a reversal.
7. The exclusion of the frontal fom the orbital margin, due to the prefrontal-postorbitofrontal contact. The prefrontal and postorbitofrontal meet in many chameleons, such as *Calumma globifer*, *Ca. ambreense* and *Ca. brevicorne* (not in *Ca. nasutum*, *Ca. fallax*, *Ca. boettgeri*, *Ca. linotum*, *Ca. vatosoa*, *Ca. guibei* and *Ca. gehringi*)^24–27^, *Chamaeleo*, *Trioceros* and it can vary among *Furcifer -* present in *F. pardalis* and *F. lateralis*, but not in *F. campani* and *F. bifidus*. Conversely, these two elements do not meet in many small chamaeleonids like *Brookesia*, *Rhampholeon* and *Rieppeleon*^24^, but also in *Bradypodion thamnobates*, *Bra. setaroi* and *Nadzikambia mlanjensis*^17^. The character optimization in Mesquite supports the prefrontal - postorbitofrontal contact as being the condition at the basal node of the clade formed by [*Chamaeleo* + *Trioceros* + *Kinyongia* + *Furcifer* + *Calumma*]. Its absence in some members of this clade can be regarded as a reversal.
8. The orbital margin, formed by the prefrontal and postorbitofrontal, is medially concave rather than straight in dorsal view. This varies among members of *Calumma*: while the margin is concave in *Ca. nasutum*, *Ca. fallax*, *Ca. guibei* and *Ca. juliae*, it is straight in *Ca. globifer*, *Ca. ambreense*, *Ca. parsonii*, *Ca. brevicorne* and *Ca. boettgeri*. Besides *Calumma*, a concave margin occurs in *Rhampholeon* and some *Furcifer* (*F. bifidus* and *F. campani*, but not in *F. pardalis* and *F. oustaleti*). It is straight in *Brookesia*, *Palleon*, *Rieppeleon*, *Archaius*, *Bradypodion*, *Trioceros*, *Kinyongia*, *Chamaeleo* and *Nadzikambia*^17,24,27–30^.
9. The sculpture formed by protuberances is well-developed and rather regularly distributed on the dorsal surface of the frontal. Among *Calumma*, an ornamentation formed by protuberances in the central region of the frontal is present in *Ca. globifer*, *Ca. ambreense*, *Ca. parsonii*, *Ca. brevicorne* and *Ca. lefona*, but not in *Ca. nasutum* or *Ca. beottgeri*^27,29,30^. Except for *Calumma*, this can be observed in *Brookesia*, *Rieppeleon* and *Bradypodion*^28,29^, but also in some large males of *Trioceros jacksonii*. Character optimization in Mesquite evaluated its presence in members of *Calumma* in two equally parsimonious ways: as the condition at the basal node in this lineage with an additional reversal in *Ca. nasutum* and *Ca. boettgeri*, or as representing a derived condition in *Ca. globifer* + *Ca. parsonii* and *Ca. brevicorne*.
10. The hour-glass shaped parietal (mid-constriction present) with a triangular wide posterior portion, which does not form a posteriorly narrowing terminus (laterally compressed crest). The parietal is flat and does not form a dorsally elevated casque. This can be observed in members of the extant *Calumma* (note that the additional widening of the posterior portion is usually less developed or absent in *Ca. nasutum*). Athough a flat parietal is present even in *Bradypodion*, *Rhampholeon* and *Rieppeleon*, it gradually narrows posteriorly, being triangular rather than hour-glass shaped. The posterior portion is usually laterally compressed, forming a crest, in *Archaius*, *Chamaeleo*, *Furcifer*, *Nadzikambia*, *Kinyongia* and *Trioceros*. *Brookesia* species and *Palleon* can be distinguished by a presence of the supratemporal processes of the parietal^15,17,24,27–29^. Character optimization in Mesquite evaluated the change of laterally compressed parietal in two equally parsimonious ways: the laterally compresed parietal as being the condition at the basal node of the [*Chamaeleo* + *Trioceros* + *Kinyongia* + *Furcifer* + *Calumma*] lineage, showing its additional reversal in *Calumma*, or the lateral compresion as representing an independent derived character in *Chamaeleo*, *Furcifer* and *Trioceros*. Character optimization in Mesquite evaluated the change of additional widening of the posterior portion of the parietal (hour-glass shape) as well as the absence of a dorsally elevated casque as being the conditions at the basal node of the *Calumma* clade.
11. The posterodorsal process of the postorbitofrontal reaches above the orbit and thus, the upper temporal fenestra is horizontal, dorsally open, being not visible in lateral view. Among *Calumma*, this is present in *Ca. globifer*, *Ca. parsonii*, *Ca. ambreense* and *Ca. brevicorne*, but not in *Ca. nasutum*, *Ca. gallus, Ca. boetgeri*, *Ca. uetzi*, *Ca. lefona*, *Ca. juliae*, *Ca. guibei* and *Ca. gehringi*^24,25,30^. The upper temporal fenestra is well visible laterally in many chamaeleonid genera, e.g. *Brookesia*, *Rieppeleon*, *Rhampholeon*, *Bradypodion*, *Furcifer* and *Chamaeleo*^24,28,29^. The polarity of this character in Mesquite is unresolved. However, the upper temporal fenestra being horizontal, dorsally open, not visible in lateral view appears to be consistently present in the lineage [*Ca. parsonii* + *Ca. globifer* + *Ca. ambreense*] sister to all other species of *Calumma*^4^.
12. The presence of a well-developed supratemporal. The supratemporal is absent in *Brookesia* and *Rieppeleon*^24^.
13. The absence of a lacrimal. The lacrimal is absent in *Calumma* (except for *Ca. parsonii*), *Rhampholeon*, *Bradypodion* and *Brookesia*, but present in *Chamaeleo*, *Kinyongia* and *Trioceros*^24^. Character optimization in Mesquite evaluated this change in two equally parsimonious ways: as the condition at the basal node in the [*Chamaeleo* + *Trioceros* + *Kinyongia* + *Furcifer* + *Calumma*] lineage with an additional reversal in *Calumma* + *Furcifer*, or as representing independent derivations of the presence of lacrimal in *Chamaeleo* and *Kinyongia* + *Trioceros*. As for the outgroup, the lacrimal is absent in *Uromastyx*, but present in *Leiolepis*^24^. The presence of lacrimal can be regarded as a plesiomorphic state among lizards^31^.
14. The partial fusion of the vomers (at least in the posterior portion). Rieppel and Crumly^24^ noted that the vomer in chamaeleons may be represented by: (a) a single fused bone; (b) an element divided anteriorly but fused posteriorly; or (c) two separate bones. Most extant chamaeleons possess a vomer that is only partly divided or fused into a single element. Paired vomers are present e.g., in *Archaius*.
15. The broad anteriormost region of the palate formed by the vomers and the anterior portions of the vomerine processes of the palatine. It is rather narrow in e.g., *Rhampoleon spectrum*, *Furcifer pardalis* and *Chamaeleo calyptratus*, but wide in *Calumma globifer*.
16. The dentary tooth row ends far anterior to the dorsal process of the coronoid^24^.
17. The presence of an angular^24^.
18. The wide skull (its width is more than 50% of the maximum anteroposterior length). It is present in the outgroup (*Uromastyx*) and character optimization in Mesquite supports wide skull as being the condition at the basal node of the clade formed by all chameleons. Character optimization in Mesquite shows that narrow, laterally compressed skull is the condition at the basal node of the clade [*Chamaeleo* + *Trioceros* + *Kinyongia* + *Furcifer* + *Calumma*], whereas the condition in *Calumma* is regarded as a reversal.
19. The absence of synapophyses on the axis. They are absent in *Calumma*, but also in *Furcifer* and *Chamaeleo*. Their presence is documented in *Rhampholeon* and *Brookesia*^32^.

### Phylogenetic placement of KNM-RU 18340

**Figure 3:**
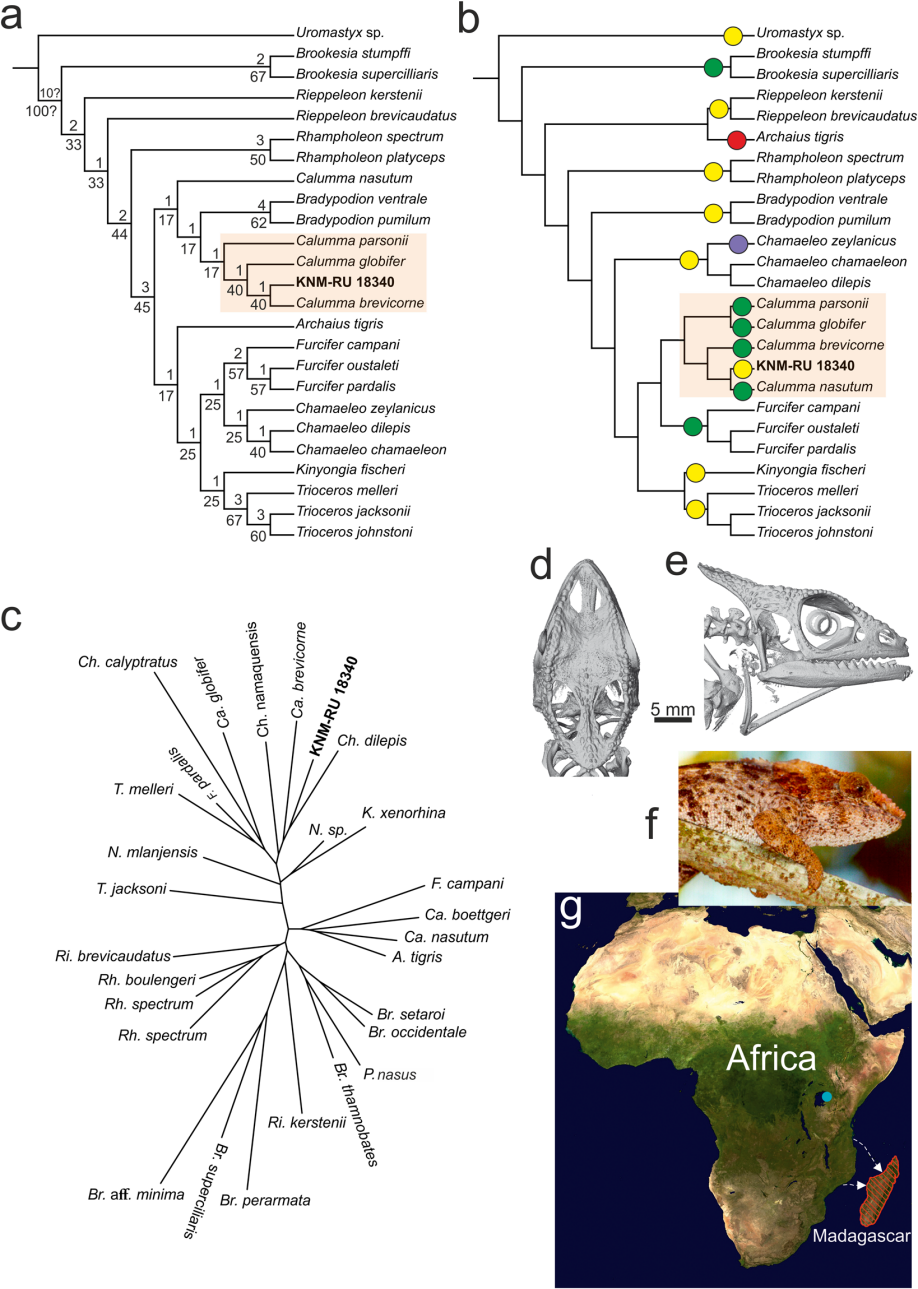
Phylogenetic position of KNM-RU 18340 (*Calumma benovskyi* sp. nov.) within Chamaeleonidae. (**a**) single parsimonious tree recovered by TNT using NT (New Technology) search (with ratchet) and 1000 iterations, showing Bremer (above node)/relative Bremer (below node) values at nodes recovered by TNT; (**b**) tree recovered by TNT using constraint based on Tolley *et al*.^4^, circles in (**b**) indicate distribution of taxa: yellow African, green Madagascar, red Seychelles and purple India. (**c**) Neighbour joining tree illustrating the phenetic similarity between the skull of the chameleons included in the analyses. The tree was constructed using the PC axes representing 99.6% of the overall variance. The analysis shows how the fossil is morphologically similar to a group of species including *Chamaeleo dilepis*, *Calumma brevicorne*, *Ch. namaquensis*, *Ca. globifer*, *Ch. calyptratus*, *Furcifer pardalis* and *Trioceros melleri*. The skull of extant *Calumma brevicorne* in (**d**) dorsal and (**e**) lateral views, and (**f**) a living representative of this species (photo by C.V.A). (**g**) Distribution of the *Calumma* species. Blue dot indicates a location of the early Miocene member *Ca. benovskyi*; red lines indicate the distribution of extant species endemic to Madagascar. White arrows indicate potential eastward dispersal routes (the map has been modified from the NASA satellite orthographic map which is in the public domain).

In all analyses (see methods), despite the different topologies within the Chamaeleoninae, specimen KNM-RU 18340 is consistently recovered as a member of the *Calumma* clade (Fig. 3a,b).

1. In analysis 1, a New Technology (NT) search in TNT produced a single most parsimonious tree. The position of KNM-RU 18340 within *Calumma* is recovered as sister to *Ca. brevicorne* (Bremer value 1, relative Bremer 40), whereas *Ca. globifer* (Bremer value 1, relative Bremer 40) and *Ca. parsonii* (Bremer value 1, relative Bremer 17) are sister to this clade (Fig. 3a). Remarkably, *Ca. nasutum* is separated from the other *Calumma* species and appears as the sister taxon of the clade *Bradypodion* + *Calumma* (the remaining species used in the analysis). According to Hillenius^33^, the skull of *Ca. nasutum* is very similar to that of *Rhampholeon*. As was already stated by Rieppel and Crumly^24^, *Ca. nasutum* lacks numerous apomorphies, possibly due to its small size.
2. In analysis 2, a traditional heuristic Traditional search in T.N.T. produced a single parsimonious tree. The topology of the examined taxa is identical to that recovered from the NT search (Fig. 3a); KNM-RU 18340 is nested within the genus *Calumma* (except for *Ca. nasutum* still recovered sister to *Bradypodion* and other *Calumma* species), as sister to *Ca. brevicorne*.
3. In the third analysis, the molecular phylogeny of Tolley *et al*.^4^ was used to constrain the ingroup relationships. The analysis produces a single tree, in which KNM-RU 18340 is again nested within *Calumma*, as sister to *Ca. nasutum* (Fig. 3b).

### Geometric morphometric analyses

Both the principal component analysis and the neighbour joining tree performed on the 3D landmarks showed that KNM-RU 18340 is morphologically most similar to a group of species including two *Chamaeleo* (*Ch. namaquensis* and *Ch. dilepis*) and two *Calumma* species (*Ca. brevicorne* and *Ca. globifer*) providing further support for the phylogenetic analysis which unambiguously places the fossil within the *Calumma* group (Fig. 3c).

## Discussion

### Taxonomic allocation of KNM-RU 18340

Rieppel *et al*.^14^ only wrote a preliminary report on the KNM-RU 18340 specimen as their study was based only on a cast and on photographs of the original material. These authors suggested that this fossil may potentionally be an early representative of the genus *Rhampholeon*, although as acknowledged by them, no living species of the genus *Rhampholeon* shows a fully separated prefrontal fenestra (called “prefrontal fontanelle” in their report) - they claimed (erroneously as we know now) the complete separation of external nares from the prefrontal fenestra in KNM-RU 18340.

Thanks to micro-computed tomography (μCT), we were able to identify all skull bones and observe their internal sides as well as their sutures. Moreover, the micro-CT revealed elements that are hidden by the rocky matrix and could not be studied before, such as an almost complete braincase with stapes, the palate region, a ceratohyal and the first three cervical vertebrae (Fig. 2, Supplementary Data 1).

The morphological comparisons are in agreement with the results of the phylogenetic analyses that specimen KNM-RU 18340 can be distinguished from all extant genera, except from *Calumma*. Overall the morphology of the fossil skull most closely resembles that seen in the extant *Ca. brevicorne* (see Fig. 3d–f), especially the shape of the parietal, the arrangement of protuberances on the parietal, the dorsal opening of the supratemporal fenestra (not visible in lateral aspect; this is also present in *Ca. parsonii*, *Ca. globifer* and *Ca. ambreense*), the presence of the prefrontal-postorbitofrontal contact over the orbit and the maximum width of the skull being located at the level of the frontal-parietal contact in dorsal view (the maximum width in *Ca. parsonii*, *Ca. globifer* and *Ca. ambreense* is posterior to this region, in the mid-section of upper temporal fenestra). But there are also some differences, e.g. the absence of the premaxilla-frontal contact, the separation of the external naris from the prefrontal fenestra, and the straight orbital margin in dorsal view in the extant species (see above).

The preserved elements of KNM-RU 18340 possess a unique combination of character states (see Diagnosis), and a new taxon name is therefore erected: *Calumma benovskyi* sp. nov. There is also one unique character: well defined and laterally expanded triangular posterolateral processes of the frontal. Although in some species of *Calumma* (e.g., *Ca. nasutum* and *Ca. vatosoa*)^24,26^ the posterolateral corners of the frontal expand laterally, they do not form a well-defined triangular processes as present in the early Miocene *Ca. benovskyi* sp. nov. The processes are not developed in *Brookesia* and *Palleon*, but are well-expanded laterally in the outgroup taxon *Uromastyx*.

### Biogeographic history of malagasy chameleons

Our results show that KNM-RU 18340 represents the first evidence of a member of the *Calumma* lineage on continental Africa. The extant species of this genus (together with the Brookesiinae, see; Fig. 3b here) are endemic to the island of Madagascar (*Furcifer* is distributed on Madagascar, but there are two species on the Comoros as well)^4,34^, which harbours an exceptional biodiversity. The separation of this island from Africa occurred already in the early Cretaceous^35^. Although the presence of a Malagasy lineage in continental Africa during the early Miocene might appear as a surprise, similar patterns have been observed for Madagascar’s endemic terrestrial extant mammals. Based on phylogenetic analyses, tenrecs^36,37^, euplerid carnivores^36,38^ and nesomyine rodents^36^ appear to be monophyletic taxa, whose sister group is found in Africa. They each result from a single colonization event and subsequent radiation within the island^39^. Likewise, recent palaeontological discoveries suggest that two lemur lineages likely dispersed from Africa to Madagascar across the Mozambique Channel independently, and thus have an African origin^36,40^. The dispersal window for these four endemic clades is estimated to span from the Oligocene to the early Miocene^36,40^. It is also worth noting that some other squamate lineages such as pythons and varanids did not manage to follow this dispersal route.

### The origin of chameleons?

Raxworthy *et al*.^3^ suggested that chameleons originated on Madagascar and dispersed over water at least three times to Africa, and once each to the Seychelles, to the Comoros archipelago (where they occupy two islands) and to Reunion. In contrast, Tolley *et al*.^4^ suggested that the family originated in Africa, with two separate oceanic dispersal events to Madagascar during the Palaeocene (Brookesiinae lineage) and the Oligocene, when prevailing oceanic currents would have favoured eastward dispersal. The early Miocene *Calumma benovskyi* sp. nov. supports an African continental origin for Malagasy chamaeleonine lineages, at least for the *Calumma* clade. The oceanic dispersal using currents that favoured eastward dispersal appears to be the most probable scenario (Fig. 3g). The existence of such eastward currents from the African shore to Madagascar between the Palaeocene and the early Miocene is well documented^41,42^. Lizards such as chameleons might have used floating islands—rafts of trees (this is especially plausible for arboreal lizards) —to cross such distances^43^. Rafting has been suggested for anole lizards migrating around the Caribbean^44^ or for mabuyid skink *Trachylepis atlantica* on the island of Fernando de Noronha (Brazil), where the ancestors of this species are believed to have rafted from Africa, across the Atlantic, during the last 9 million years^45^; *Trachylepis* is otherwise distributed in Africa and Madagascar^46^.

The African origin of *Calumma* is also supported by the distribution of members forming a clade [*Chamaeleo* + *Trioceros* + *Kinyongia* + *Furcifer* + *Calumma*]; where only the latter two taxa are found in Madagascar^4^. According to Tolley *et al*.^4^, *Calumma* and *Furcifer* are sister taxa and the split of these two lineages occured in the Eocene. But within these genera, species-level divergence occured during the Oligocene and Miocene. However, if the split is older than the documented early Miocene occurrence of the extinct continental species *Ca. benovskyi* sp. nov., this might suggest that the last common ancestor of these two lineages most likely lived on the continent. This then suggests a continental origin for both, *Calumma* and *Furcifer*.

Although the Miocene *Calumma benovskyi* sp. nov. represents the oldest known member of this genus and shares some characters with the basal node of this clade, based on our current knowledge we can assume that *Ca. beniovskyi* most likely does not form an ancestral lineage for the *Calumma* clade. It is recovered as sister to *Ca. brevicorne* rather than in a basal position to all *Calumma*. Moreover, molecular data suggest that the ancestral lineage should be older than the early Miocene^4^; yet, this remains to be verified with fossil data of an older age. For now the diversity and morphological disparity of the Oligocene and early Miocene *Calumma* in Africa remains unknown. However, the assignment of KNM-RU 18340 to the genus *Calumma* and its presence in the early Miocene of Kenya demonstrates that the *Calumma* lineage was present in Africa at a time when oceanic currents supported eastward dispersal^41,42^. This is consistent with the previously suggested hypothesis of Tolley *et al*.^4^ based on molecular data.

### Is the alternative scenario possible?

The above scenario challenges a dispersal of the genus *Calumma* from Madagascar to Africa through oceanic dispersal, as previously suggested by Raxworthy *et al*.^3^. According to several authors (e.g., Ali and Huber^41^, Samonds *et al*.^42^), the prevailing oceanic currents at that time support dispersal from Africa to Madagascar, and not the other way around. According to McCall^47^, some areas of the Mozambique Channel were dry land during the middle Eocene - early Miocene. Even if animals used several intervening islands in a stepping-stone chain, eastward currents would have hindered their westward dispersal. Moreover, chameleons are relatively poor swimmers rendering the westward route scenario even less probable. Ali and Huber^41^ also pointed out that shortly after the early Miocene, the currents between Africa and Madagascar turned in the opposite direction (i.e. westwards, toward Africa), like in present-day surface-water circulation. From the middle Miocene onwards, currents would thus have hindered journey to Madagascar for any non-volant and non-swimming taxa, and could have supported the development of insular endemism for terrestrial animals there.

Hence, the “out of Madagascar” dispersal scenario would have required the existence of fully terrestrial land bridge between the late Oligocene and the early Miocene. Of note is that the separation of Africa and Madagascar occurred already during the Mesozoic^35^. But such a bridge would have enabled any taxon, even large animals (e.g., elephants), to disperse. This scenario would neither explain the quasi absence of large-bodied terrestrial mammals, nor the limited number of mammal families that live on the island today.

## Methods

### Material

The skull KNM-RU 18340 (field number RU 1916'87) is housed at the National Museums of Kenya, Nairobi. The specimen comes from the early Miocene locality Rusinga Island and is remarkably complete and three-dimensionally well-preserved. Its maximum anteroposterior length (from the snout to the end of parietal) is 32.1 mm. The specimen has not been fully prepared from the rock and this matrix still fills the whole internal region and hides many bony elements.

### X-ray microtomography and three-dimensional visualization

We µCT scanned this skull at the University of Witswatersrand (South Africa), using a Nikon Metrology XTH 225/320 LC Xray microtomograph, with an acceleration tension of 100 kV and an intensity of 50 µA. As much as 2000 radiographs were acquired with an exposure time of 4 s and an averaging of 1. The resolution was 18.83 µm. The micro-CT data were analyzed using VG Studio Max 3.2 and Avizo 8.1. The right side of the skull is in better condition and more completely preserved than the left side. For this reason, we focused our reconstruction efforts on the right side, and supplemented certain areas with elements from the left side, when better preserved. For the reconstruction of the whole skull in dorsal view, missing areas were restored by mirror imaging.

### Phylogenetic analysis

A morphological data matrix (see Supplementary Data 2) was developed based on characters taken primarily from Rieppel and Crumly^24^ (see Supplementary Data 1). Nineteen characters of relevance for chameleons were added. The matrix comprises 43 characters scored for 23 extant chameleon ingroup taxa (*Archaius tigris*, *Bradypodion ventrale*, *Bra. pumilum*, *Brookesia superciliaris*, *Bro. stumpffi*, *Calumma globifer*, *Ca. parsonii*, *Ca. brevicorne*, *Ca. nasutum*, *Chamaeleo zeylanicus*, *Ch. chamaeleon*, *Ch. dilepis*, *Furcifer campani*, *F. oustaleti*, *F. pardalis*, *Kinyongia fischeri*, *Rhampholeon spectrum*, *Rh. platyceps*, *Rieppeleon kerstenii*, *Ri. brevicaudatus*, *Trioceros melleri*, *T. johnstoni* and *T. jacksonii*), in addition to the extinct taxon represented by the Kenyan material described here. *Uromastyx* was used as the outgroup. The principal goal of this analysis is to understand the relationship of the Kenyan early Miocene taxon among Chamaeleonidae. The data matrix was analysed using maximum parsimony as an optimality criterion in the program TNT and the NT (New Technology) search (with ratchet) with 1000 iterations^48^. All characters were treated as unordered and were equally weighted. Support was estimated through Bremer support indices^49^. Mesquite v.2.75^50^ was used to visualize all trees. In the third analysis (see above), the molecular phylogeny of Tolley *et al*.^4^ was used to constrain the ingroup relationships. The command used was: [force = ((1 2) (((14 (3 4))) ((((5 6) ((8 9) ((17 (16 15)) ((((18 (19 20))((12 13) (10 11)))((7 ((23 (21 22)))); constrain=;]. KNM-RU 18340 was the only taxon excluded from the constraint tree, leaving it free to float.

### Geometric morphometric analyses

We ran a 3D geometric morphometric analysis on 27 species of chameleons in addition to the fossil. Extant species covered all genera with most genera represented by two or three species. Specimens were micro CT-scanned and surfaces were cleaned in Geomagic Studio and imported in the ‘Landmark’ software^51^. Forty-three landmarks (see Supplementary Data 1 - Supplementary Tab. 1) were taken on the right side of each skull to describe the overall shape (Supplementary Data 1 - Supplementary Fig. 17). A general Procrustes analysis (GPA)^52^ was run and was followed by a principal component analysis (PCA) performed on the Procrustes residuals. We constructed a neighbor-joining tree on the PC axes that jointly explained 99.6% of the variance to evaluate phenotypic proximity between the fossil and the extant genera. All analyses were performed in R (v. 2.15.3; R Core Team 2016) using the libraries RMORPH^53^, MASS^54^, APE^55^ and ADE4^56^.

### Character reconstruction

Mesquite v.2.75^50^ was used for optimizations and ancestral state determinations using parsimony (see Supplementary Data 1 - Supplementary Fig. 18). The Mesquite tree is based on Tolley *et al*.^4^. KNM-RU 18340 is excluded here due to its uncertain topology within *Calumma*. In contrast, *Ca. boettgeri* is added to increase taxon sampling in regards of *Calumma*.

## Supplementary information

Supplementary Information.

Supplementary Information2.

## Data Availability

Digital surface models of the figured fossil specimen KNM-RU 18340 are available on the Dryad Digital Repository (10.5061/dryad.msbcc2fts). The following items are available in this collection: KNM-RU 18340, holotype: 3D printable surface file in STL format.
