# Supplementary material for: The only complete articulated early Miocene chameleon skull (Rusinga Island, Kenya) suggests an African origin for Madagascar’s endemic chameleons
Source: Sci Rep. 2020 Jan 10;10:109. doi: 10.1038/s41598-019-57014-5 (PMC6954250; doi:10.1038/s41598-019-57014-5)
Supplement: Supplementary file 2 — Supplementary Information2. [file 41598_2019_57014_MOESM2_ESM.pdf]

Supplementary Data 1

Contents:

1. Geological setting

2. Detailed description and virtually isolated elements of *Calumma benovskyi*

3. Specimens examined

4. Data matrix

5. Geometric morphometric analyses

6. Character Reconstruction

7. References

1. Geological setting

Rusinga Island (0°24'S, 34°0'E) is an island located in Kenya at the eastern edge of Lake Victoria, in the Nyanza Rift system, a failed arm of the main Great African Rift. The island is also situated on the outer edge of the Kisingiri Volcano, which was active during the Miocene. The early Miocene deposits on Rusinga record this volcanism, the chemistry of which plays a major role in the exceptional fossil preservation on the island[1-6]. The majority of the fossils found on Rusinga come from the Rusinga Group succession, which consist of pyroclastic, fluvial, lacustrine and lahar deposits[7]. This succession is (from oldest to youngest): the Wayondo Formation, Kiahera Fm., Rusinga Agglomerate, Hiwegi Fm., and Kulu Fm.[3,7].

According to Rieppel *et al.*[8], the fossil described in this paper was found at site R107 (approximately 0° 25' 30" S,34° 9' 0" E) in the Hiwegi Fm. However, R107 exposes the contact between the Rusinga Agglomerate and the Hiwegi Fm. Yet, the former is not known to bear any fossils, and the grey-green matrix adhering to the specimen points to the Hiwegi Fm. It is probable, therefore, that the specimen comes from the base of the Hiwegi Fm. Based on K-Ar dates, Drake *et al.*[9] suggested that the Hiwegi Formation was about ~17.9 Ma old, and that the entire fossiliferous Rusinga Group sequence was deposited during less than half a million years. However, more recent analyses using 40Ar/39Ar dates, magnetostratigraphy, and lithostratgiraphy showed that the fossiliferous layers on Rusinga were deposited over a much longer time interval, between ~17-20 Ma, and that the base of the Hiwegi Fm. was approximately 18 Ma old[7,10-12].

It has been long assumed that the entire Rusinga Group represents a relatively stable (i.e., unchanged through time) paleoenvironment[13-15]. However, recent studies have shown evidence of paleoenvironmental variability, ranging from open, dry woodland habitats to

dense closed-canopy forest [11],[16-20]. Paleobotanical and sedimentological data indicate a warm climate with seasonal precipitation[17].

2. Detailed description and virtually isolated elements of *Calumma benovskyi*

Dermal skull roof

Premaxilla. The premaxilla is straight and small (Supplementary Fig. 1a-c). This rod-like element is narrow along its entire length. It widens only slightly laterally at its anteroventral half as a results of the presence of maxillary facets on each side. The posterodorsal end of the premaxilla reaches the frontal and together they prevent the mutual contact of the right and left nasals.

Maxilla. The description is based on the complete right maxilla. The bone is rather elongated and relatively massively built with a slight medial curvature at its anterior end (Supplementary Fig. 1d-g). The external surface of the bone is smooth. The perpendicular facial process increases dorsally. This process is divided by a large and rounded external naris into a larger posterior portion and a smaller anterior portion. Both portions are not dorsally connected to one another, thus the external naris is not separated from the prefrontal fenestra. In lateral view, the posterior portion of the facial process is slightly constricted in the midregion. A flat and mediolaterally broad premaxillary process is located medially to the anterior portion of the facial process. Posterodorsally, the premaxillary process overlaps the anterior region of the nasal. In the medial view, a prominent medial horizontal expansion is visible. It forms the palatine process of the maxilla, bearing a large facet for the palatine on its dorsal side. The horizontal expansion runs from the front of the maxilla to the level of the 4th

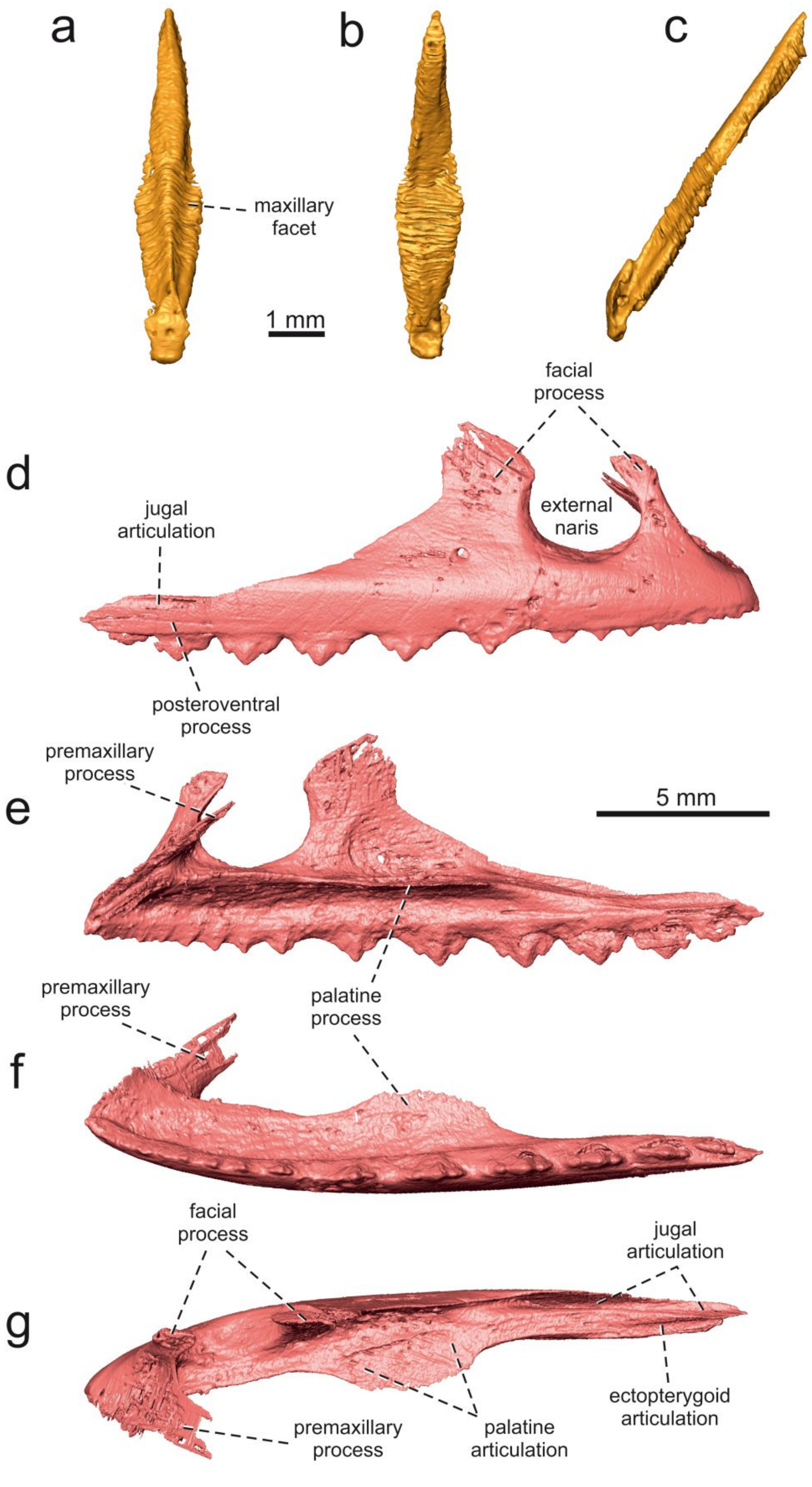

Supplementary Fig. 1 *Calumma benovskyi* sp. nov., the holotype KNM-RU 18340 from the lower Miocene of Kenya. Virtually isolated premaxilla in **a** anterior; **b** posterior; and **c** lateral views. Virtually isolated right maxilla in **d** lateral; **e** medial; **f** ventral; and **g** dorsal views.

tooth (counted from posterior) , where it merges gradually with the bone. An essentially straight supradental shelf supports a single row of 15 teeth. Tricuspidity starts from the 9th tooth (counted from anterior). On the dorsal surface, the posterior portion of the posteroventral process of the maxilla bears a concave and deep articulation area for the jugal. The internal wall of this articulation is higher than the external one in this posterior region.

Nasals. The nasals are paired, and both are preserved. They are flat and elongated elements, but small relative to the overall size of the skull (Supplementary Fig. 2a-d). The right and left nasals have no mutual medial contact as they are separated by a strut of bone formed by the contact of the premaxilla with the anteromedian process of the frontal. The anterior portion of the nasal bears the facet for the maxilla anterodorsally, and it is overlapped by the frontal in the posterior region. Thus, only mid-portion of the nasal is exposed externally. This region partly forms the lateral margin of the prefrontal fenestra.

Prefrontal. Both prefrontals are preserved. In lateral view, the prefrontal is a roughly triangular element, protruding posterodorsally into a broad posterodorsal process (Supplementary Fig. 2e-f). This process is flat and its lateral margin forms the anterodorsal portion of the orbit. The medial margin of the process is in contact with the frontal, whereas a short contact with the postorbitofrontal is present posteriorly. This contact excludes the frontal from taking a part in the orbital margin. The anterolateral margin of the prefrontal curves slightly posterodorsally, bearing here a prominent sculpture. The sculpture consists of four simple aligned protuberances. The two protuberances located in the middle are large, whereas the first and last one are distinctly smaller. The rest of the surface is rather smooth. The surface of orbital lamina is large and medially deep. The internal surface is excavated for the nasal capsule. In the posteroventral region, a large articulation facet for the palatine is present.

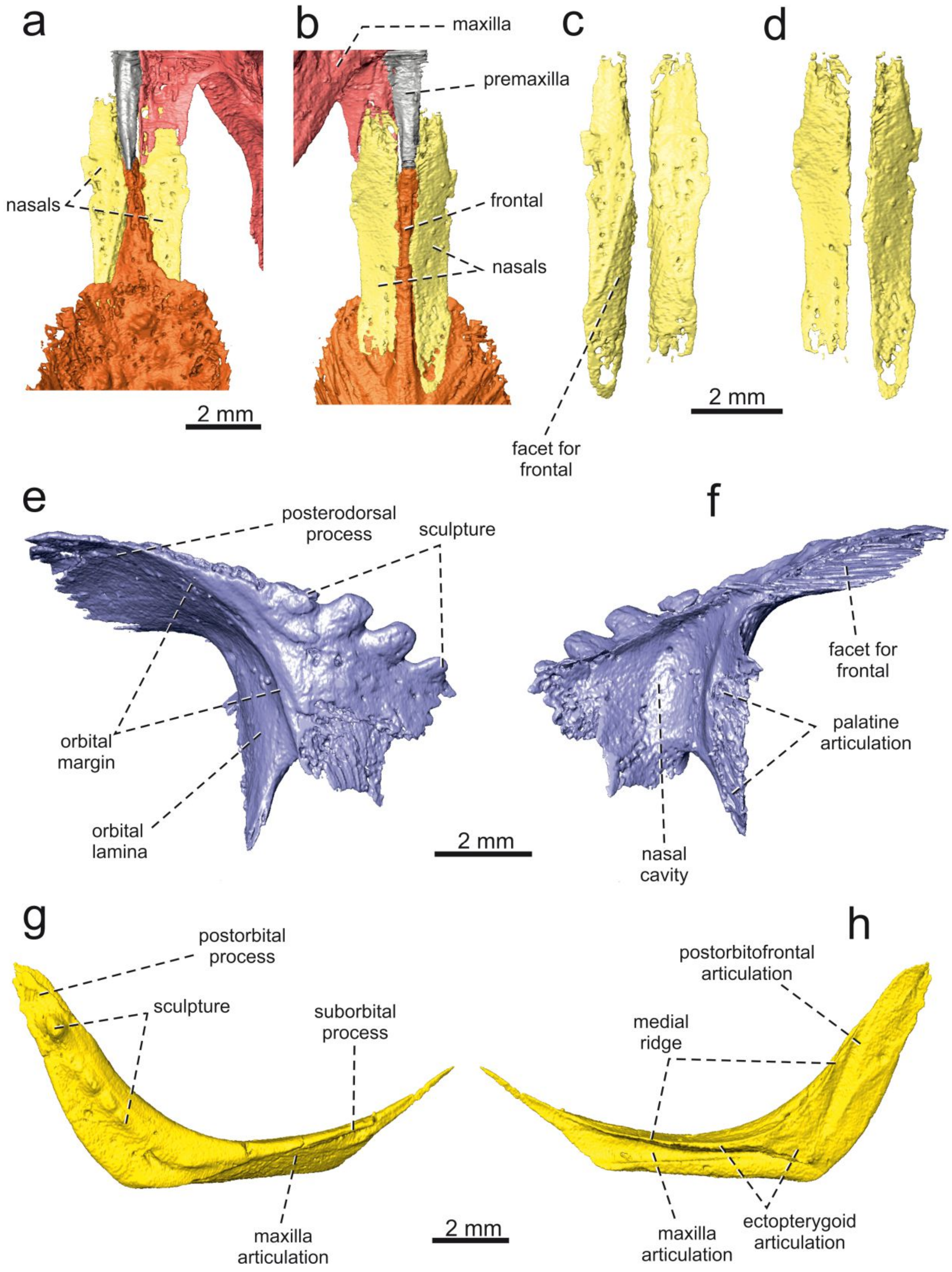

Supplementary Fig. 2 *Calumma benovskyi* sp. nov., the holotype KNM-RU 18340 from the lower Miocene of Kenya. Details of the nasal region in **a** dorsal; and **b** ventral views. Virtually isolated nasal in **c** dorsal; and **d** ventral views. Note the absence of contact between right and left nasals. Virtually isolated right prefrontal in **e** lateral; and **f** medial views. Virtually isolated right jugal in **g** lateral; and **h** medial views.

Jugal. The jugals are preserved on both sides, but the right one is in better condition. This element is crescent-shaped and can be divided into two processes: a suborbital process and a postorbital process (Supplementary Fig. 2g-h). They form the posteroventral and posterior margins of the orbit. The jugal exhibits facets for the maxilla, postorbitofrontal and ectopterygoid. The postorbital process is slightly wider than the suborbital process, although it gradually tapers dorsally. This process bears a sculpture along its external mid-region. It consists of several protuberances arranged in a single line and more strongly developed in the dorsal region. These protuberances more-or-less decrease in size ventrally. The internal surface of the postorbital process bears a large facet for the postorbitofrontal, which becomes narrower ventrally and slightly disappears. The suborbital process of the jugal is longer than the postorbital one, protruding anteriorly into a very narrow process. Its external surface is largely covered by the maxilla, such that only the dorsalmost portion of the suborbital process is exposed at its lateral aspect. At the internal aspect, the jugal possesses a well developed medial ridge of the type 3 (sensu Čerňanský *et al.*[21]). Since the ventral portion of the jugal is wedged in the maxilla, an articulation surface with the latter is present on this side as well. Dorsally to it, the articulation with the ectopterygoid is present. It approximately mirrors the course of the medial ridge.

Frontals. The frontals are fused to a single element. It is large, forming the dominant element of the skull roof. It is excluded from the orbit by the contact of the prefrontal and postorbitofrontal. The frontals are elongated and relatively broad, widening posteriorly to form the wedge-shaped posterolateral corners (Supplementary Fig. 3). At the level of these triangular posterolateral processes, the bone is pierced in its central region by a small, anteroposteriorly elongated foramen. Anteriorly to this region, the element only slightly

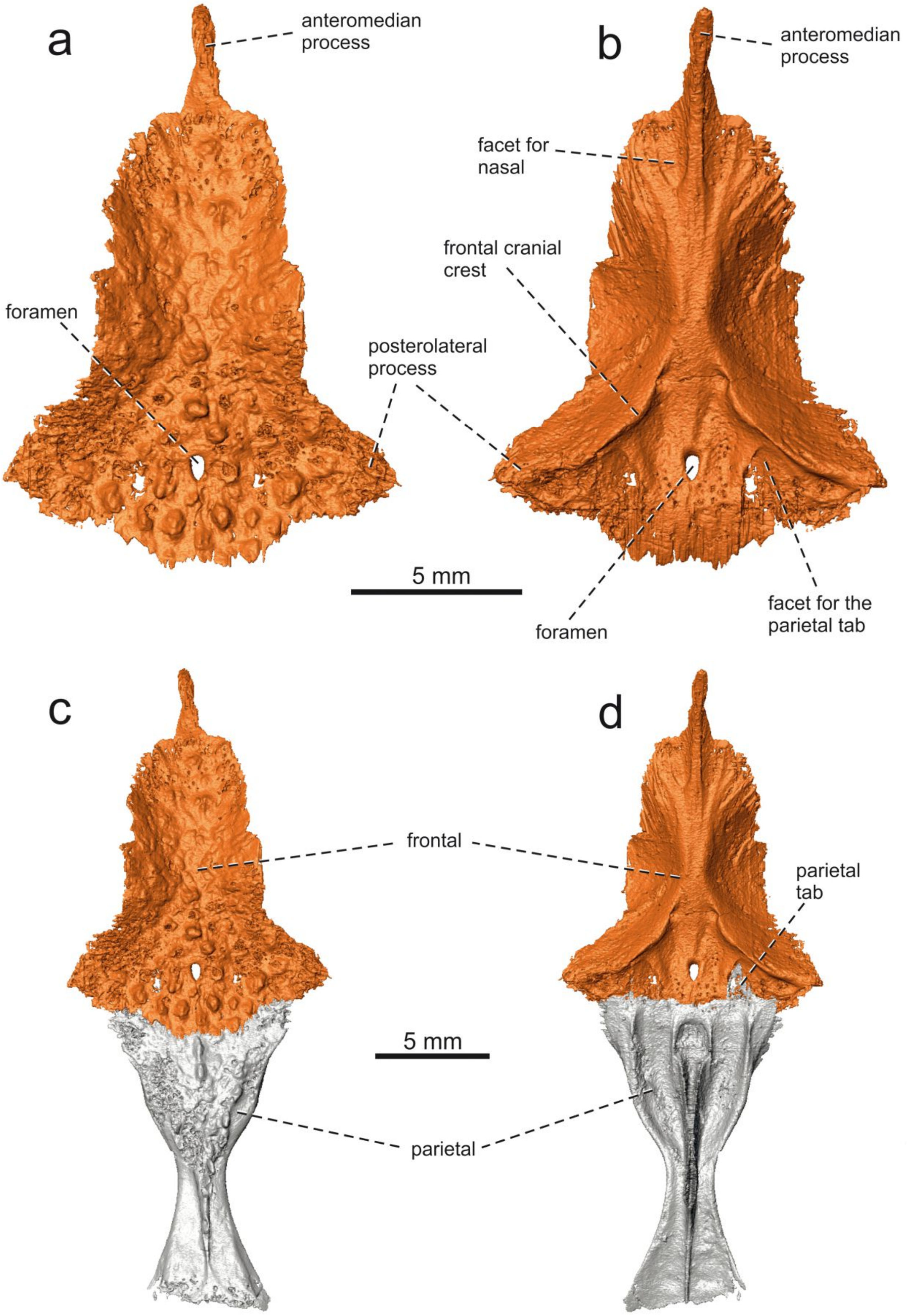

Supplementary Fig. 3 *Calumma benovskyi* sp. nov., the holotype KNM-RU 18340 from the lower Miocene of Kenya. Virtually isolated frontal in **a** dorsal; and **b** ventral views. Virtually isolated frontal and parietal in connexion in **c** dorsal; and **d** ventral views.

narrows. However, the anterior-most portion of the bone is markedly extended into a thin but distinct anteromedian process. The central region is somewhat depressed, whereas the lateral margins of the frontal are slightly inclined dorsally. A well-developed ornamentation is formed by protuberances. They are moderately spaced and rather regularly distributed, covering the otherwise smooth external surface. Toward the posterior margin of the bone, the protuberances become gradually bigger and anteroposteriorly elongated. In the anterior half of the bone, they are less distinct.

The internal surface of the fused frontals bears two distinct frontal cranial crests, being strongly developed especially at the posterior half of the bone. Here, they are sharp, running posterolaterally toward the lateral end of the posterolateral processes. Medially and posteromedially to this region, a large wedge-shaped facet for the parietal tab is located at both sides. These facets are associated with the posteriorly slightly expanded central portion of the frontal. This portion overlaps the anterior region of the parietal. In the anterior region, the crests converge close to each other, running almost parallel anteriorly. In this region, they are, however, less defined. A very shallow groove (or longitudinal depression) can be found between them. The bony portions lateral to these crests are flat. In the anterior region, two ovoid and moderate sized facets for the nasals are located. They are separated by a strong central ridge, which extends the anteromedian process of the frontal.

Parietal. The parietal is an anteroposteriorly elongated element, that is constricted in its mid-length. This gives the entire parietal an hourglass shape, but with the anterior portion being wider than the posterior one (Supplementary Fig. 4). The main sculpture of the dorsal surface of the parietal is formed by protuberances arranged in three lines - the central one and two laterally located ones (although only the right branch is completely preserved). At the level of

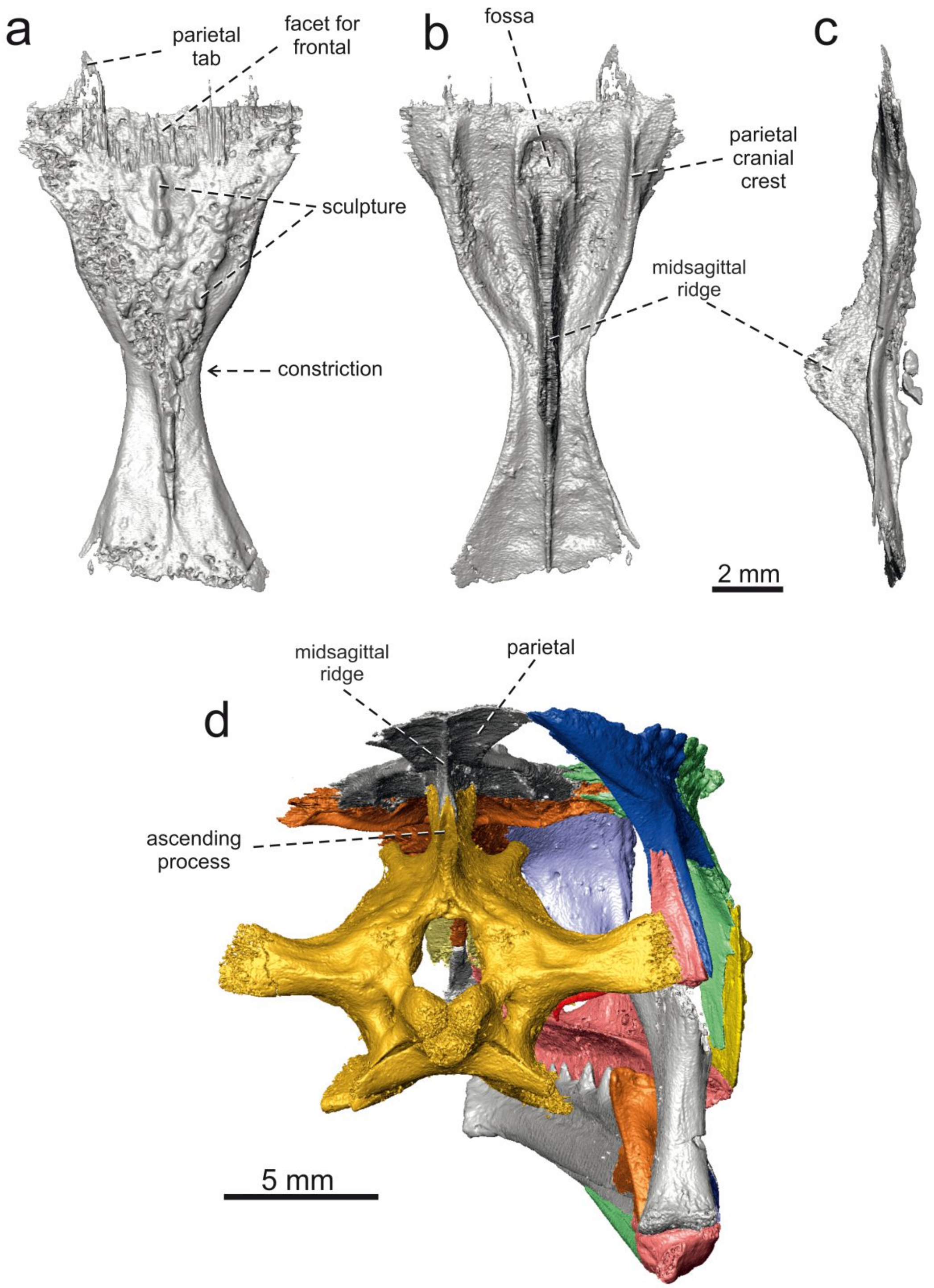

Supplementary Fig. 4 *Calumma benovskyi* sp. nov., the holotype KNM-RU 18340 from the lower Miocene of Kenya. Virtually isolated parietal in **a** dorsal; **b** ventral; and **c** lateral views. Virtually segmented bones from the dorsal and right side (best preserved elements) in **d** posterior view. Note the contact between parietal and the ascending process of the supraoccipital.

the parietal mid-constriction, these three lines merge together and form a single central ridge that runs further posteriorly. At the posteriormost preserved portion, it gradually disappears. Thus, this sculpture arrangement roughly resembles the Greek letter Ψ (psi). The anterior region of the dorsal surface of the parietal bears a large, posteriorly concave facet for the frontal - the frontal overlaps this area of the parietal. This contact is strong, because there are also two large triangular parietal tabs, which protrude anteriorly. However, only the left one is well identified (the right one being poorly preserved).

The internal region of the parietal is dominated by the midsagittal ridge which forms a contact with the supraoccipital. In lateral view, the ridge is rounded, reaching the deepest ventral level in the area of the parietal mid-constriction. In this region and anteriorly to it, the ridge is divided into two branches by a deep longitudinal groove. The dorsal portion of the ascending process (the processus ascendens) of the supraoccipital crest fits into this groove, forming a firm articulation. Anteriorly, the midsagittal crest becomes low and the groove expands into a rounded fossa. Posteriorly, the midsagittal ridge runs to the end of the preserved portion of the bone. However, it is very low here and not divided by a groove; this region does not play a role in the contact with supraoccipital. The parietal cranial crests start posteriorly to the level of the lateral margin of the parietal tabs. They run posteriorly to the lateral margins of the bone, where they gradually merge with them.

Postorbitofrontal. The postfrontal and postorbital are fused to form the postorbitofrontal. It forms the posterior and posterodorsal margin of the orbit (Supplementary Fig. 5a. b). This element has an irregular shape, but three processes can be identified. The ventral process gradually tapers, forming the contact with the jugal, whereas the ventral portion of the process runs beneath the jugal. The line of protuberances from the jugal continues on the dorsal aspect

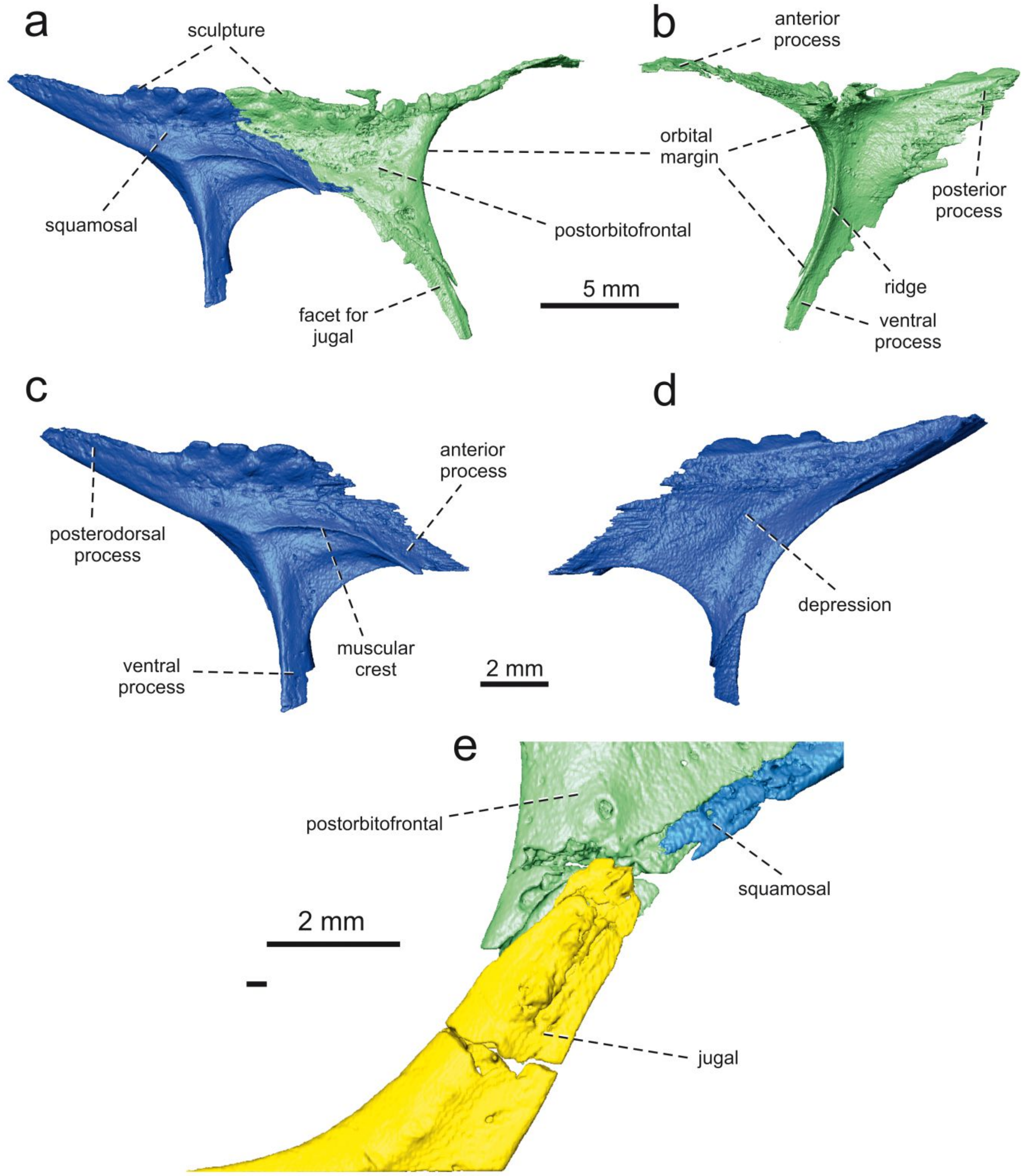

Supplementary Fig. 5 *Calumma benovskyi* sp. nov., the holotype KNM-RU 18340 from the lower Miocene of Kenya. Virtually isolated right squamosal and postorbitofrontal in connexion in **a** lateral view. Virtually isolated right postorbitofrontal in **b** medial view. Virtually isolated right squamosal in **c** lateral; and **d** medial views. Details of the left orbital region in **e** lateral view. Note the position of the squamosal close to the jugal-postorbitofrontal contact.

of this ventral process. It consists of three or four small protuberances (better preserved on the left side) positioned in a single curved line, running somewhat in parallel to the anteriorly located orbital margin. The posterior process of the postorbitofrontal is narrow in dorsal view, but wide in lateral view. Here, it forms a firm contact with the squamosal. It overlaps the squamosal laterally. This process bears a distinctly developed sculpture running along its dorsolateral margin. The sculpture is formed by protuberances arranged in two lines. The dorsal line is represented by large, dorsally directed protuberances. The protuberances located in the posterior region are anteroposteriorly elongated. The line located immediately ventral to it is formed by weaker protuberances. The protuberances of both lines alternate with each other in lateral view, thus their mutual position forms a zipper-like structure. The dorsal line continues to the dorsolateral margin of the long anterior process, but the protuberances become less distinct here and are not elongated. Medially, this process forms a table with a rounded (anteriorly concave) posterior margin. This margin forms the anterior border of the upper temporal fenestra. The process contacts here the anterolateral process of the parietal and further anteriorly, it contacts the posterior half of the frontal. The anterior end of the process is in contact with the prefrontal. A medial ridge, a prolongation of the one on the jugal, runs on the internal aspect along the anterior margin of the ventral process of the postorbitofrontal. It forms the posterodorsal border of the orbit. The surface posterior to the ridge is depressed, forming a cavity. The articulation for the ectopterygoid is present in the ventral region of the ventral process.

Squamosal. The right squamosal is nearly completely preserved. It is a triradiate element (Supplementary Fig. 5c, d). The posterodorsal process is long, rather narrow, and gradually curves medially. Its posterior end contacts the parietal and thus closes the large upper

temporal fenestra. The dorsal margin of the central region of the squamosal bears a sculpture. This sculpture is in continuation with the one of the postorbitofrontal and is formed by a line of large dorsally located protuberances and a line of ventrally located small protuberances. The dorsal line is formed by three elongated protuberances, which gradually decrease in size posteriorly. The anterior process of the squamosal is triangular. It forms a facet that is wedged into the posterior margin of the postorbitofrontal. It is not in contact with the jugal, although it should be noted that on the left side, the squamosal lies close to the jugal (Supplementary Fig. 5e). On the lateral surface of the bone is a sharp, dorsally convex muscular crest. The ventral process is a stem-like structure, bearing the facet for the supratemporal on the ventral half of its medial surface. The ventral end of the process joins the lateral portion of the quadrate. In lateral view, a ridge runs along the entire lateral surface of the process and gradually narrows ventrally. Between this ridge and the muscular crest is a bony septum with a rounded margin.

The internal surface of the squamosal bears a wedge-shaped depression, which narrows posteriorly. It is framed by a more-or-less straight ridge dorsally and by a sharp rounded ridge posteroventrally.

Supratemporal. It is a small but robustly built element (Supplementary Fig. 6a-d). It is wedge-shaped and gradually widens ventrally bearing here a robust contact area with the quadrate. The supratemporal is located on the medial side of the ventral portion of the squamosal. The ventral portion of the supratemporal forms the contact with the quadrate.

Quadrate and palate region

The palatal region is preserved, although somewhat damaged (Supplementary Fig. 7). There is no dentition present on the palate.

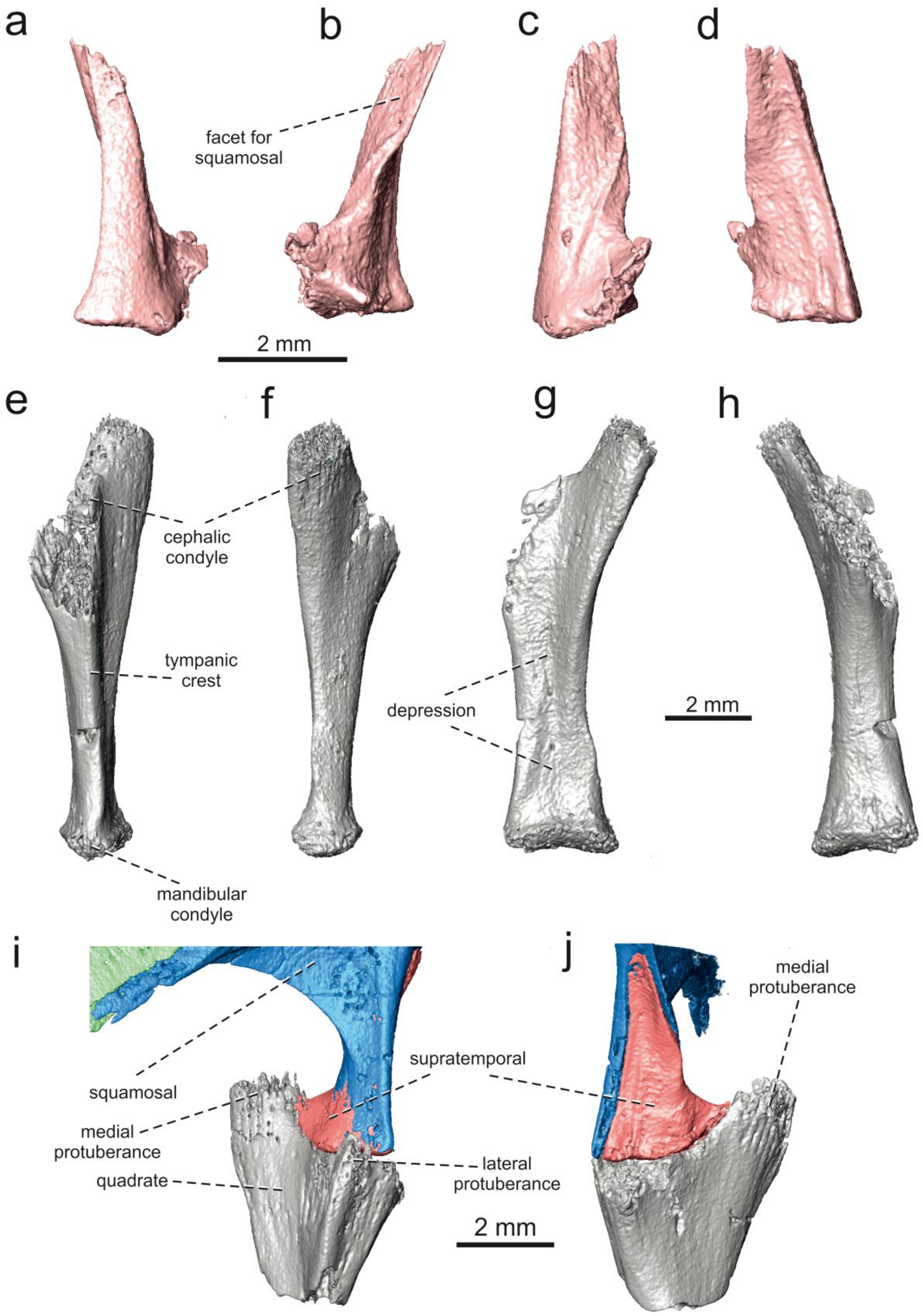

Supplementary Fig. 6 *Calumma benovskyi* sp. nov., the holotype KNM-RU 18340 from the lower Miocene of Kenya. Virtually isolated right supratemporal in **a** lateral; **b** medial; **c** anterior; and **d** posterior views. Virtually isolated right quadrate in **e** lateral; **f** medial; **g** anterior; and **h** posterior views. Details of the left otic region in lateral **i** lateral and **j** posterior views. Note the broad contact between quadrate and supratemporal.

Quadrate. A more-or-less complete quadrate is available on the right side (Supplementary Fig. 6e-h), whereas only the dorsal portion of the left quadrate is preserved (Supplementary Fig. 6i, j). In lateral view, the quadrate is dorsoventrally elongated and quite narrow. It gradually widens dorsally. The dorsal portion forms a cephalic condyle, which is divided into two parts. The lateral protuberance is lower, whereas the medial protuberance is higher and medially inclined. The concave area between the two protuberances forms the contact with the squamosal and the supratemporal. The ventral portion of the quadrate gradually tapers (both in lateral and medial views). The straight tympanic crest runs laterally along the entire central region of the quadrate. The quadrate is wide in posterior aspect and this side possesses a centrally located shallow depression. The mandibular condyle is mediolaterally wide and appears to be rather concave (best seen in posterior and anterior views).

Vomer: The vomers lie in the anteromedian portion of the palate (Supplementary Fig. 7). They are small and only partly preserved. The posterior regions of the right and left vomer are partially fused together, but the bad preservation of the anterior region makes it difficult to evaluate the extent of that fusion. Both vomers form a flattened element, ovoid in shape (in ventral/dorsal view), with slightly laterally expanded, convex margins. The anteriormost portion contacts the premaxilla and maxilla. On the dorsal side, the vomers bear two symmetrical longitudinal ridges running along the mid-region. The ridges continue on the vomerine processes of the palatine. The ridges are slightly concave medially, being closest to each other at the level of the palatine-vomer contact. This area is also the widest section of the whole element in dorsal view. The two ridges however, do not meet and there is thus a space forming a groove between them. There is no vomer-pterygoid contact.

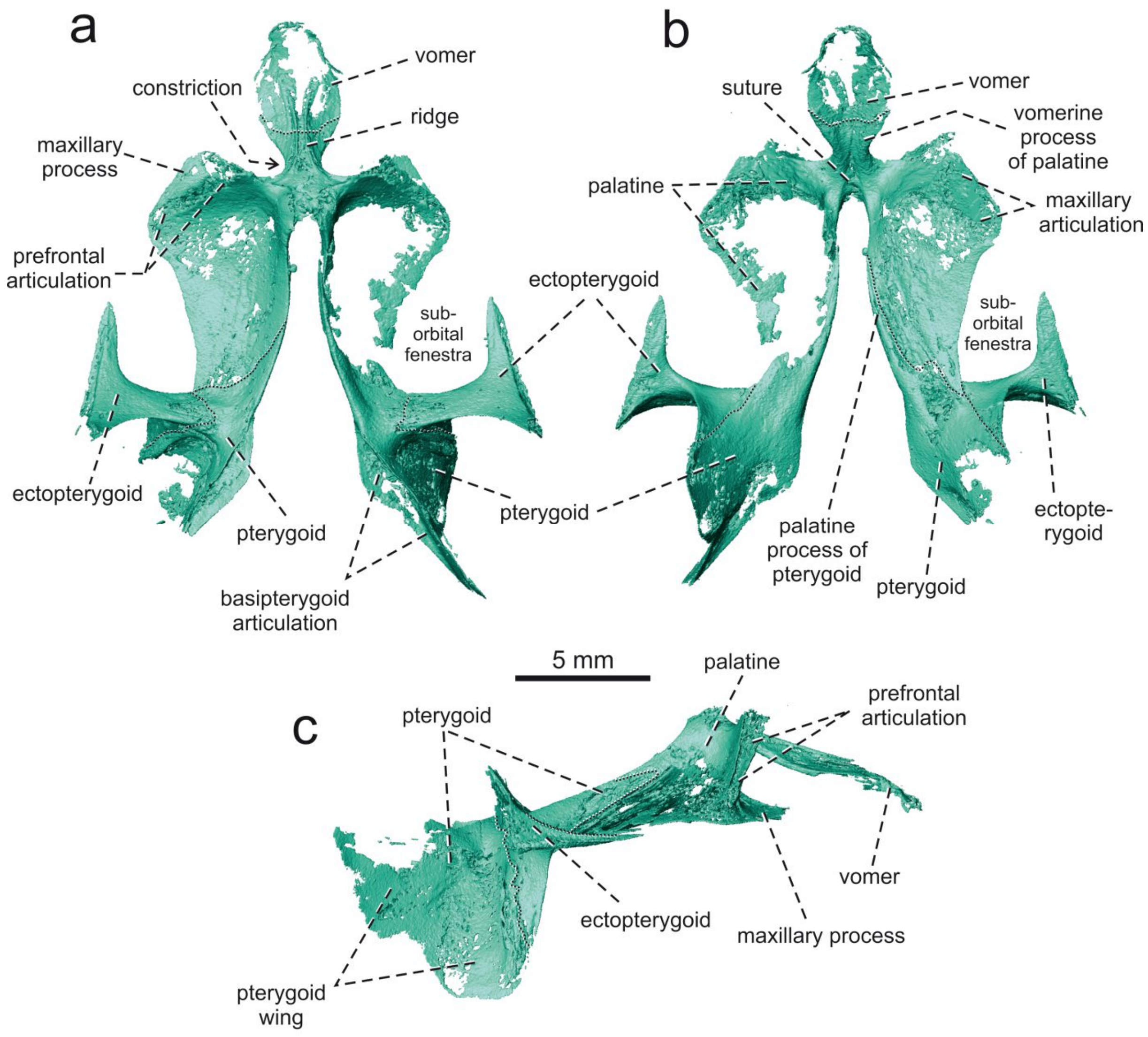

Supplementary Fig. 7 *Calumma benovskyi* sp. nov., the holotype KNM-RU 18340 from the lower Miocene of Kenya. Virtually segmented palate in **a** dorsal; **b** ventral; **c** lateral views.

Palatine: The palatine is a paired bone. The left one is more-or-less completely preserved, whereas the right one is largely damaged. There is only a short anterior contact between left and right palatine that is marked by a distinct, sinuous suture. Further posteriorly, both palatines are separated by a gap and their median margins run parallelly for some distance. Overall, the palatine is a large and flat bone. The anterior region is divided into three different portions: the vomerine process, the maxillary process, and the articulation with the prefrontal. The well-developed vomerine process is the anterior-most part of the palatine, and left and

right sides are in contact here. In lateral view, the vomerine processes are aligned with the vomer but are at an angle (about 130°) with the rest of the palate. The processes show a strong constriction in the posterior half of their length and gradually widen anteriorly. In ventral view, and posterior to the constriction, a groove (or trough) is present along the mid-region. The maxillary process lies laterally and ventrally from the vomerine one, and forms the anterior ventral region of the palate. It bears a large horizontal articulation facet for the maxilla, which overlaps the palatine process of the maxilla. Dorsal to it, there is a strong articulation for the prefrontal that is anterodorsally inclined, forming an angle of 70° with the horizontally lying facet for the maxilla.

The posterior main portion of the palatine is sail-like shaped and narrows gradually posteriorly. In cross-section, this wide and flat portion of the bone bends slightly dorsally in the medial direction. Thus, the lateral margin, which forms the margin of the large suborbital fenestra, lies more ventrally relative to the medial margin of the palatine. This medial margin forms a sharp, dorsally elevated edge that is in contact with the pterygoid. This contact is more exposed in ventral aspect and reaches the level of the anterior border of the suborbital fenestra. The posterior portion of the palatine is bifurcated in ventral view, having a shorter posteromedial and a longer posterolateral process. The posterolateral one forms a short contact with the ectopterygoid.

Ectopterygoid: Each pterygoid is a posteromedially-anterolaterally oriented and forms a bridge between the pterygoid posteromedially and the ventromedial margin of the cheek anterolaterally. It borders the suborbital fenestra posterolaterally. Besides the pterygoid, the ectopterygoid contacts the maxilla, the jugal, and the postorbitofrontal. The ectopterygoid is an axe-shaped element consisting of two portions – a lateral and a medial portion. The lateral

portion is wider and has a longer anterior region than the posterior one. The medial portion, which contacts the pterygoid, is slightly twisted compared to the lateral one.

Pterygoid: The pterygoid is a paired and broad element. It lies in the posterior portion of the palate. The left and right pterygoids are widely separated by the interpterygoid vacuity. Here, their medial margins are shallowly concave. The pterygoid has a long and thin palatine process, mainly exposed in the ventral region. Posterior to its contact with the ectopterygoid, the pterygoid forms a large, but thin, ossified pterygoid wing. It is posteroventrally expanded, being heart-shaped in lateral view. Its anteromediodorsal region possesses an articulation with the basipterygoid processs. There is no contact with the quadrate.

Braincase

The stapes is preserved only on the right side (Supplementary Fig. 8), but the braincase is almost completely preserved (Supplementary Fig. 9).

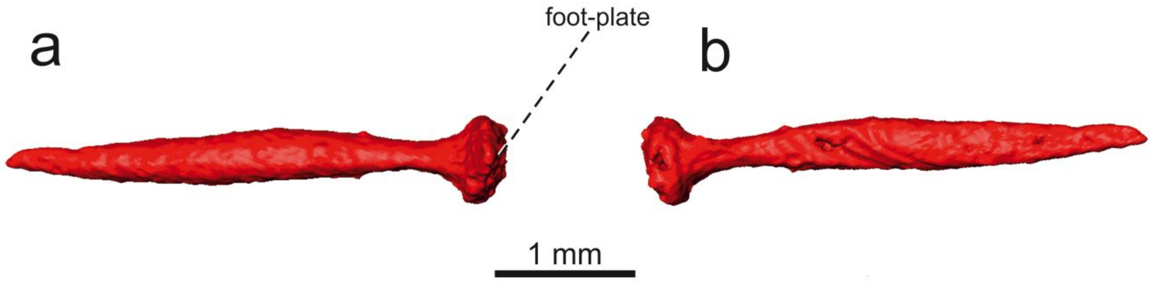

Supplementary Fig. 8 *Calumma benovskyi* sp. nov., the holotype KNM-RU 18340 from the lower Miocene of Kenya. Virtually isolated right stapes in **a** dorsal; and **b** ventral views.

Stapes. Only the right stapes is preserved (Supplementary Fig. 8). It is a small, laterally elongated, straight and needle like element. Its medial portion is located in the oval window of the braincase (fenestra ovalis, Supplementary Fig. 9a) and forms the foot-plate. It is a rounded

and knob-like structure. From the foot-plate, the bone becomes thinner laterally, but is again slightly thicker in its mid-portion, until the lateral termination tapers into a point. There is no stapedial foramen.

Supraoccipital. The unpaired supraoccipital forms the dorsal portion of the braincase, the dorsal part of the inner ear capsule, and the dorsal margin of the foramen magnum (Supplementary Fig. 9a-c, e, f). It has a high ascending process with which the ventral crest of the parietal articulates. In dorsal view, the ascending process is broadest and more robust in the anterior region, whereas it gradually narrows posteriorly. Ventrolaterally to it, a pair of smaller anterolateral processes are present in the anterior region. The supraoccipital consists also of two laterally expanded ventrolateral bony portions, which give the bone a subrectangular shape in dorsal view. The dorsal surface of these lateral portions expands posterolaterally to contact the otooccipital and then becomes narrower toward the foramen magnum. The posterior border of the supraoccipital is sinuous and forms the anteriorly concave margin of the foramen magnum. In the anterior view, the supraoccipital is inverted letter Y-shaped. In this view, the medial opening of the endolymphatic foramen is visible in the ventral region of the inner side of the supraoccipital.

Prootic. The prootic is a paired element forming the dorsolateral border of the braincase (Supplementary Fig. 9a-c). It contacts the supraoccipital, otooccipital, basisphenoid, and basioccipital. An anteriorly strongly expanded alar process is absent - the anterior margin of the prootic is slightly sinuous in lateral view. The lateral side of the prootic bears a well-developed and sharp prootic crest (crista prootica) oriented posterodorsally. A foramen for a facial nerve opens ventromedially from the prootic crest. In the internal side of this area, a large foramen for the vestibulocochlear nerve is present. The posteroventral portion of the

prootic forms the anterior margin of the oval fenestra where the foot plate of the stapes is placed.

Otooccipital. The otooccipitals are the units formed by co-ossification of the opisthotics and exoccipitals (Supplementary Fig. 9f). The fusion area is marked by the vagus foramen (Supplementary Fig. 9b). Immediately next to it is a small foramen for the hypoglosal nerve (Supplementary Fig. 9h). The otooccipital forms the posterior mid-region of the braincase, the lateral borders of the foramen magnum and 2/3 of the occipital condyle in its dorsolateral region. These two portions of the occipital condyle are in contact and consequently exclude the basioccipital from the foramen magnum. The otooccipitals contacts the quadrate, supratemporal, prootic, supraoccipital, and basioccipital. The paroccipital processes are large and laterally expanded. The anterior portion of the otooccipital forms the internal posterior wall of the auditory capsule. Ventral to this, the medial wall of the cavum cochleare is pierced by the perilymphatic foramen.

Basioccipital. The basioccipital is a broad, unpaired element, forming the posteroventral portion of the braincase (Supplementary Fig. 9D, F). The overall shape is roughly rhomboidal in ventral view. Its posterior portion forms the ventral 1/3 of the occipital condyle. The occipital condyle itself, when including the two otooccipital parts, is heart shaped and its maximum width equals its maximum height. In dorsal view, the condyle has anteriorly concave margin. The basal tubercles of basioccipital are ventrolaterally oriented and short.

Sphenoid. Anterior to the basioccipital, the sphenoid is present and the suture between these two bones is still well-visible (Supplementary Fig. 9D). The suture is not straight, but resembles the letter M. The sphenoid is a midline unpaired element, forming the anteroventral

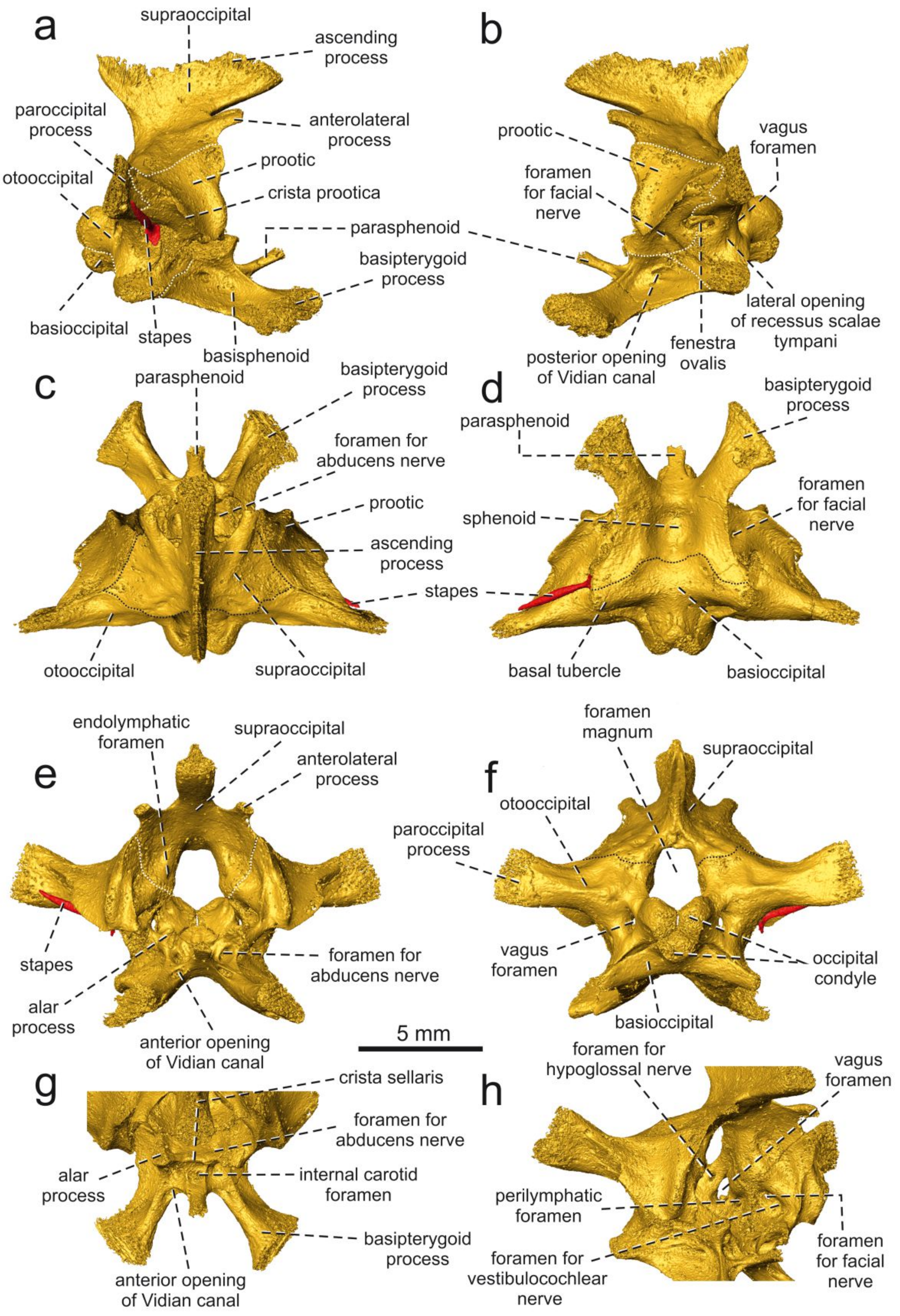

Supplementary Fig. 9 *Calumma benovskyi* sp. nov., the holotype KNM-RU 18340 from the lower Miocene of Kenya. Virtually segmented braincase in **a** right; **b** left; **c** dorsal; **d** ventral; **e** anterior; and **f** posterior views. Details of the anteroventral region of the sphenoid in **g** anterodorsal view. Detail of the left internal region of the braincase with foramina in **h** dorsoanterolateral view.

portion of the osseous braincase and the connection between the palate and the braincase. The contacts with pterygoids consist in the large, robust anterolaterally oriented basipterygoid processes. Anteriorly, the mid-portion of the sphenoid protrudes into a short, but distinct parasphenoid process. In ventral view, the central region of the sphenoid bears a deep and rounded fossa. Laterally, close to the root portion of basipterygoid process, the bone is pierced by the posterior opening of the Vidian canal (Supplementary Fig. 9e, g). Anteriorly, this canal opens lateral to the base of the parasphenoid process. The anterior foramen for the abducens nerve is located directly above the anterior opening of the Vidian canal. A pair of the internal carotid foramina are located centrally, dorsal to the base of parasphenoid process. A sharp crista sellaris is found dorsal to these foramina and runs between the well-developed alar processes. Medially to the alar process, the dorsal surface is pierced by the posterior foramen for the abducens nerve.

Mandible

Dentary. The dentary is elongate, forming the dominant component of the mandible (in lateral view, it is four times longer than the postdentary portion of the mandible; Supplementary Fig. 10). The dentary is slightly convex laterally at its dorsal aspect and mediolaterally compressed. In lateral view, the height of the dentary gradually increases posteriorly. The straight subdental shelf bears 13 teeth. Ventrally to it, the dental groove is present, being shallow rather than deep. The ventral portion of the dentary in medial view bears a deep Meckel's groove for Meckel's cartilage. It is roofed by a well developed straight supra-alveolar ridge and floored by a slightly thickened ventral margin of the dentary. Meckel's groove gradually narrows anteriorly and turns ventrally at the level of the 6th tooth (counted from anterior). The alveolar foramen is located at the level of the penultimate tooth. The symphysis is well developed, but narrow. The posterior region of the dentary forms two

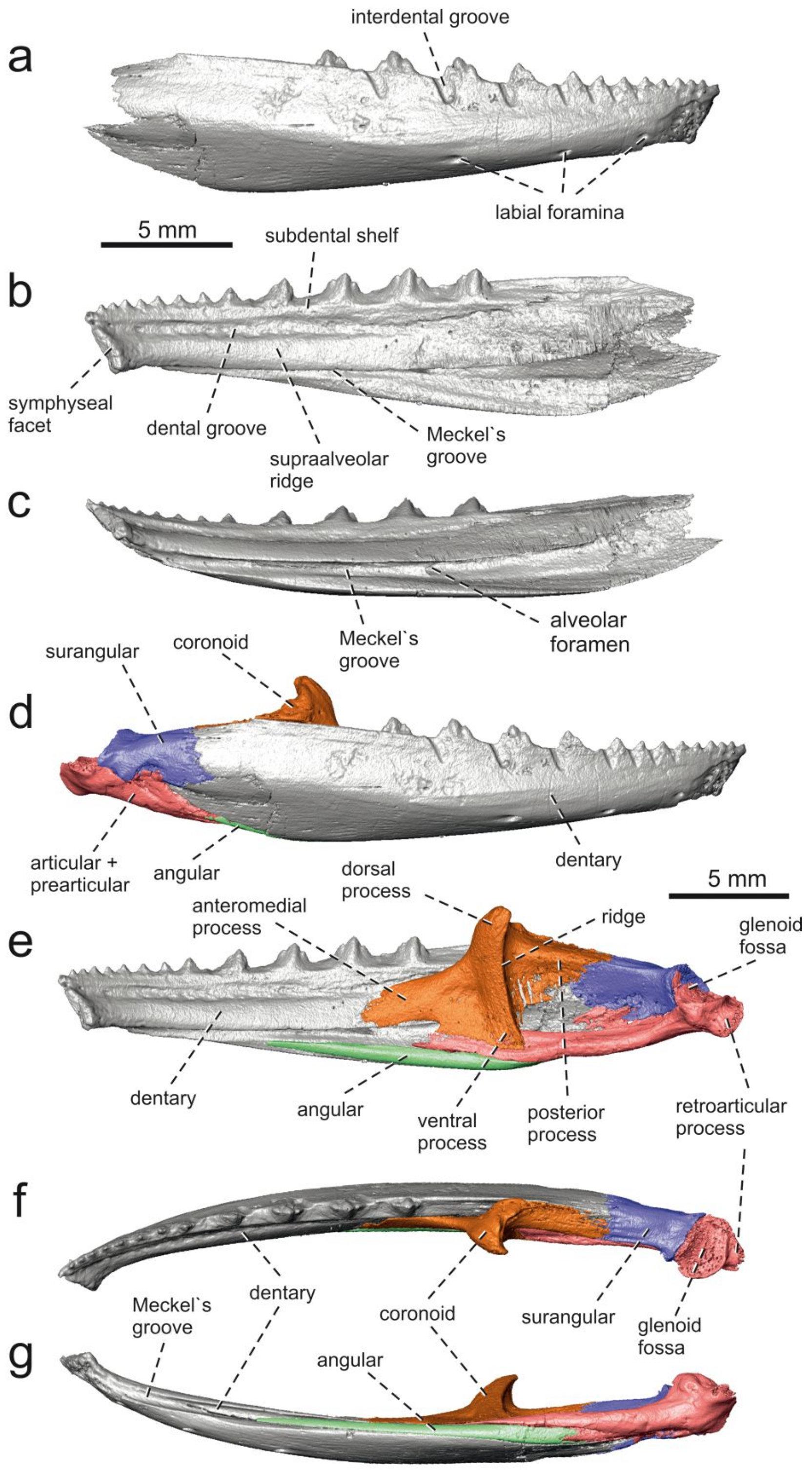

Supplementary Fig. 10 *Calumma benovskyi* sp. nov., the holotype KNM-RU 18340 from the lower Miocene of Kenya. Virtually segmented right dentary in **a** lateral; **b** medial; and **c** ventromedial views. Virtually segmented complete right mandible in **d** lateral; **e** medial; **f** dorsal; and **g** ventral views.

distinct, posteriorly directed triangular processes. The ventral process reaches further posteriorly than the dorsal one. The otherwise smooth lateral surface of the dentary is pierced by three labial foramina located close to the ventral margin. Furthermore, along its dorsal margin, the lateral surface bears well-developed triangular interdental grooves.

Coronoid. The coronoid is a cross-shaped bone, lying in the posterior half of the mandible. It has four processes: the dorsal, posterior, anteromedial, and ventral process. The dorsal process lies in the mid-length of the bone and it is exposed also from the lateral aspect. It is slightly bent posteriorly. Although it is shorter than the anteromedial or posterior processes, its overall appearance is robust. Its posteromedial portion bears a distinct ridge (or keel) for muscle attachment. The posterior process is equally long as the anteromedial one. It is triangular, gradually narrowing posteriorly. Here, it overlaps the contact of the dentary with surangular medially and slightly dorsally, but not laterally. The anteromedial process expands anteriorly, covering the posterior portion of the supra-alveolar ridge. The process is blunt, reaching the level of the penultimate tooth. The ventral process is short, but broad. It forms the contact with the prearticular + articular. The coronoid has no contact with the angular.

Angular. The angular is well-developed, separated from other bones of the mandible. It is an elongate, rod-like element lying along the ventral margin of the lower jaw. The anterior portion of the angular is straight and protrudes. It gradually narrows to a point. It contacts the posteroventral portion of the dentary and reaches the level between 3rd and 4th tooth position. In lateral view, however, only a short portion of the angular is exposed.

Surangular. Only the right surangular is more-or-less completely preserved. The surangular is a short, but massive element forming the posterodorsal portion of the mandible. It is

roughly rectangular in cross-section. At the posterior end, the bone is slightly elevated dorsally and forms the anterior border of the glenoid fossa.

Articular + prearticular. These two bones appear to be fused in the specimen redescribed herein and form the posteroventral portion of the mandible. The prearticular is thin and protrudes anteriorly forming a process. It reaches the level of the posterior most tooth. This portion forms a dorsally concave trough, where the posteroventral portion of dentary fits. The articular is short and robust, forming a glenoid fossa for the quadrate on its dorsal surface. In dorsal view, the glenoid fossa is medially enlarged. The retroarticular process, located posterior to the fossa, is short and posteroventrally directed.

Dentition

Teeth are present on both the maxillae and the dentaries. Tooth implantation is acrodont. The six posterior-most teeth of the tooth row on the maxilla and four on dentary are more strongly developed than the rest, which diminish in size anteriorly. The teeth, especially the ones located in the posterior region of the tooth row, are strongly tricuspid. The central cusp is dominant, being about twice the size of its mesial and distal accessory cusps. The distal cusp is slightly larger than the mesial one on the upper teeth, whereas it is the other way around for the lower teeth. The teeth are compressed mediolaterally. The sizes of the inter-dental gaps are small in the anterior region and distinctly widen posteriorly. Thus, the posterior large teeth have large interdental gaps and their bases are not in contact. On the posterior region of the maxilla, the size of the inter-dental gaps decreases again. The last posterior two maxillary teeth are slightly inclined from the axis of the tooth row, a condition that slightly resembles the imbricate alignment of the last teeth mentioned by Čerňanský (2011). The tooth row in dentary ends anterior to the dorsal process of coronoid.

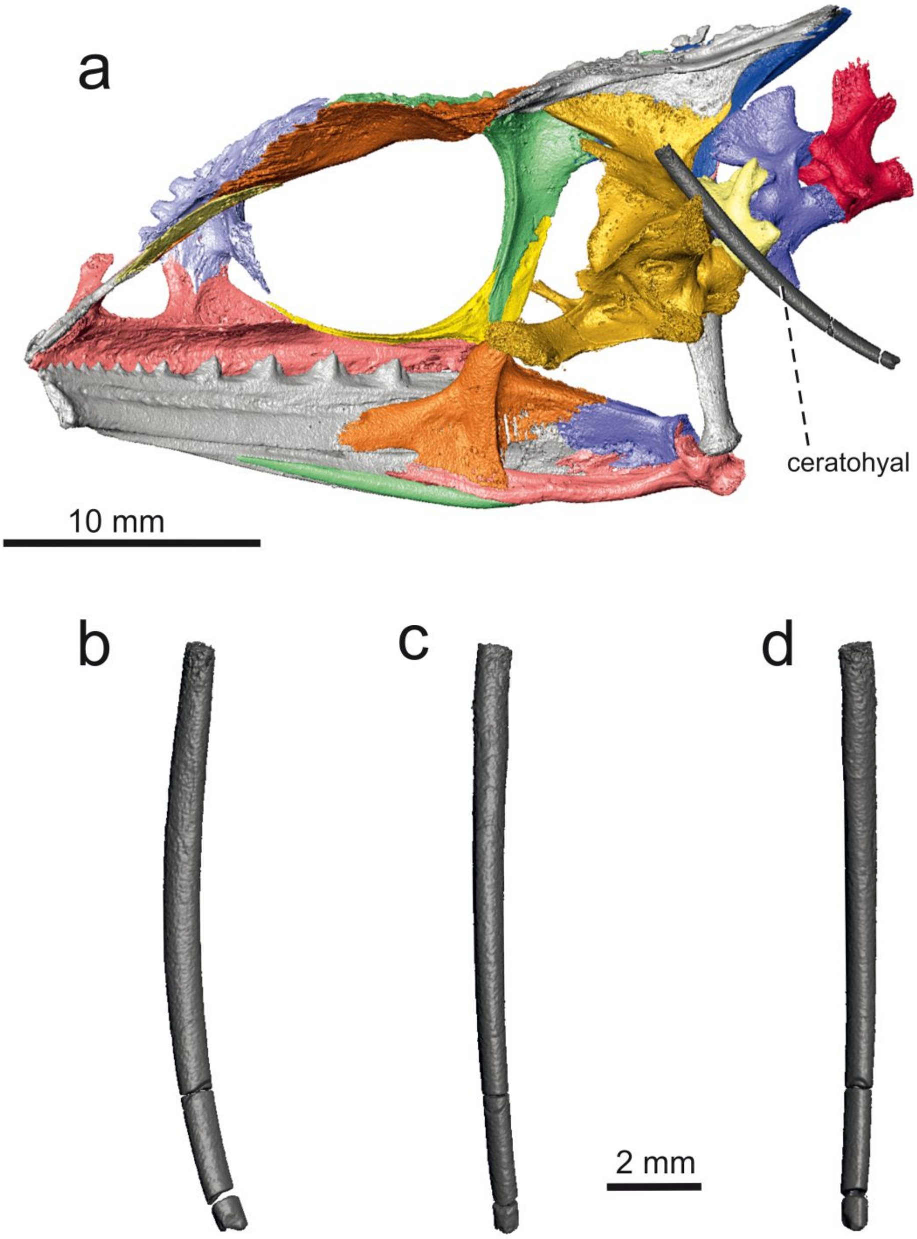

Supplementary Fig. 11 *Calumma benovskyi* sp. nov., the holotype KNM-RU 18340 from the lower Miocene of Kenya. Virtually segmented internal region of the skull based on the dorsal and right side (best preserved elements) in **a** medial view. Note the in situ position of the left ceratohyal in the skull. Virtually isolated left ceratohyal in **b** lateral; **c** anterior; and **d** posterior views.

Hyoid

Ceratohyal. The long, rod-like element visible on the left side is interpreted here as a ceratohyal (Supplementary Fig. 11). The element is straight in anterior and posterior aspect, and very slightly narrows ventrally. In lateral view, it is however slightly bent, convex anteriorly.

Postrcanial

Atlas. The atlas of chamaeleonids is composed of three parts that are united: the two halves of the atlantal neural arch and the ventrally located first intercentrum (Supplementary Fig. 12). However, the intercentrum is not preserved here. The neural arch consists of paired pedicles and paired laminae. The laminae are straight in anterior view, forming a triangular structure. In lateral view, they are anteriorly inclined, wide, but short relative to the height of the neural spine of the axis. The dorsal margin of the laminae is rounded. The anterior margin is continuous, but the posterior margin suddenly widens at mid-length and continues to the posterodorsal process. The posterodorsal process is markedly dorsally inclined, bearing a postzygapophyseal facet on its ventromedial side. The process is pointed, but does not precede the zygapophyseal articular facet posteriorly.The ventrally located pedicles are robust. The robust transverse processes are well laterally expanded, having a posterior inclination. They are blunt-ended. The anterior region of this ventral region of the atlas bears two articular surfaces for the occipital condyle. The posterior region bears articular surfaces for the axis.

Axis. The axis is overall dorsoventrally high, mostly due to the tall neural spine. The latter has a fan-shaped extension dorsally and forms a rounded (convex) dorsal margin. Here, the neural

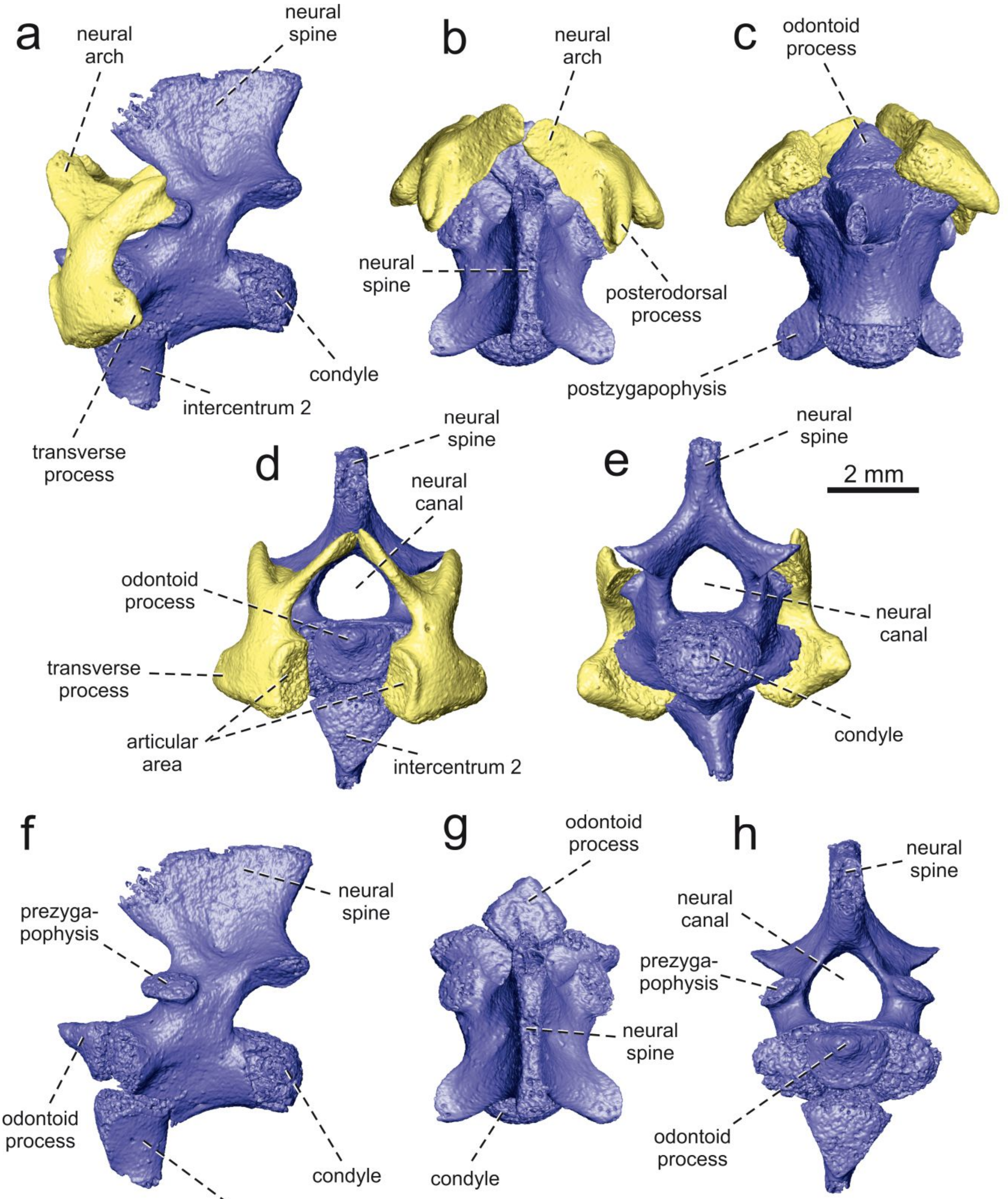

Supplementary Fig. 12 *Calumma benovskyi* sp. nov., the holotype KNM-RU 18340 from the lower Miocene of Kenya. Virtually segmented atlas-axis complex in **a** lateral; **b** dorsal; **c** ventral; **d** anterior; and **e** posterior views. Virtually isolated axis in **f** lateral; **g** dorsal; and **h** anterior views.

spine is slightly extended and projects more anteriorly, whereas the posterior 2/3 of the dorsal margin is less rounded. In dorsal view, the neural spine has robust appearance, being slightly constricted in its mid-region. The prezygapophyseal articular facets are rather small. They are elliptical, slightly prolonged anteroposteriorly and inclined ventrolaterally (around 30° from the horizontal plane). The postzygapophyses are laterally expanded and slightly inclined posteriorly. They possess oval articular surfaces. The neural canal is medium-sized, almost pentagonal in shape. Its height is approximately 22% of the entire vertebra. The synapophyses are absent. The odontoid process, which is formed by an atlantal pleurocentrum, is incompletely fused in this specimen.The process is anteriorly directed and protrudes visibly. It fits into the atlantal ring anteriorly. The condyle is large, almost rounded. The anteriorly located ventral margin of the centrum is concave in its lateral aspect. In anterior direction, it gradually declines ventrally. In ventral view, the lateral margins of the body are slightly concave. A pair of small foramina is located in mid-region of the centrum. The intercentrum is large. It is triangular and is fused tothe centrum with traces of this fusion still visible. The intercentrum is oriented ventrally.

Third cervical vertebra. The vertebra is high relative to its length (Supplementary Fig. 13). The neural spine is smaller and exhibits a different morphology in comparison to that of the axis. The neural spine starts to rise gradually dorsally, being narrower and posteriorly inclined. It is tall and roughly rhomboidal in shape. In dorsal view, the neural spine is thin - its thickness is largest at its mid-region. However, its dorsal margin is damaged. The prezygapophyses are well developed and bear anteriorly oriented articular facets. The postzygapophyses are large. The neural canal is roughly pentagonal. The synapophyses are present, laterally expanded and slightly posteriorly inclined. They reach the furthest lateral level of all the vertebral projections. The cotyle and condyle are incompletely preserved, but

both appear to be slightly depressed. The ventral margin of the centrum is concave. The intercentrum is not preserved.

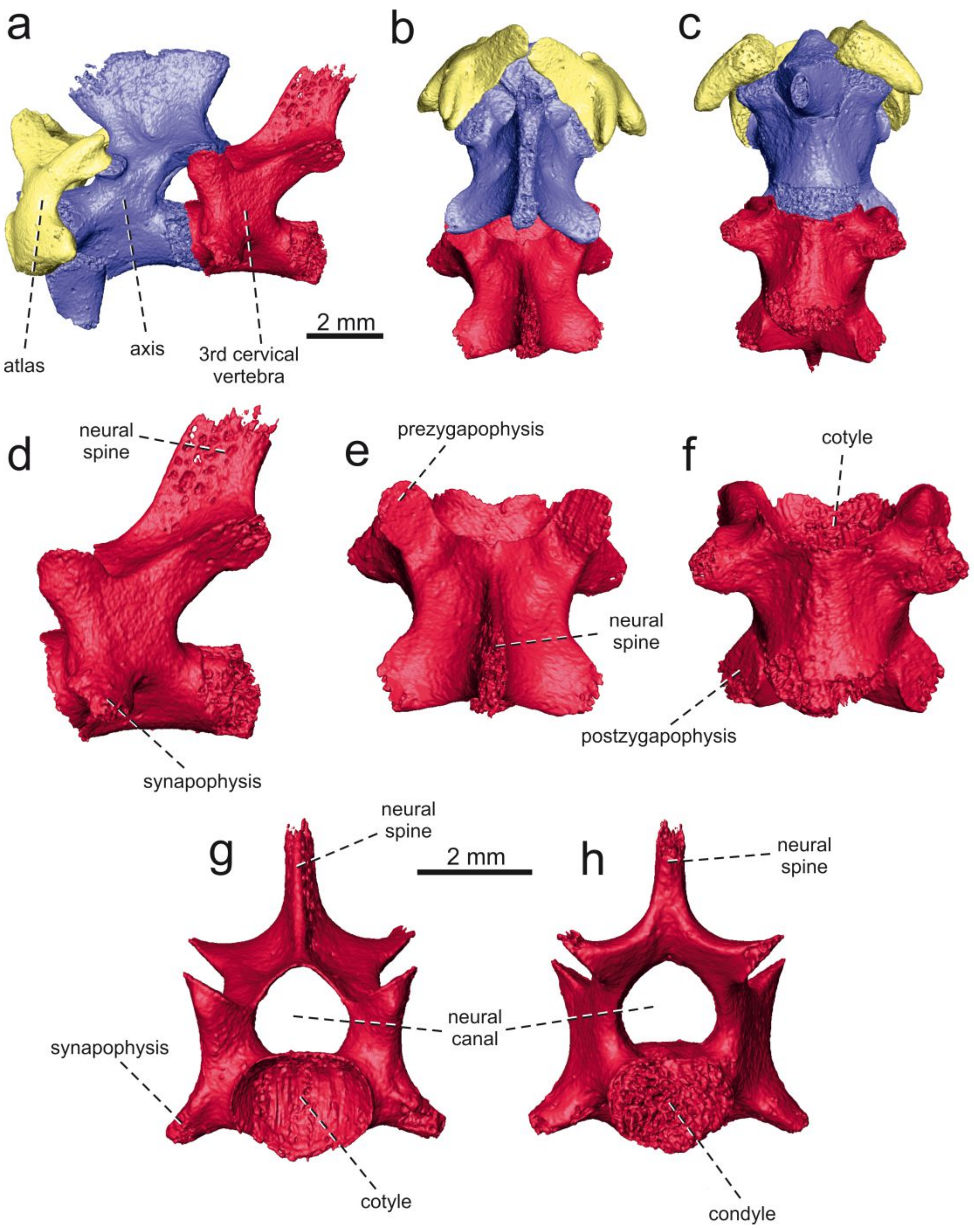

Supplementary Fig. 13 *Calumma benovskyi* sp. nov., the holotype KNM-RU 18340 from the lower Miocene of Kenya. Virtually segmented cervical region with the three first cervical vertebrae in **a** lateral; **b** dorsal; and **c** ventral views. Virtually isolated 3rd cervical vertebra in **d** lateral; **e** dorsal; **f** ventral; **g** anterior; and **h** posterior views.

3. Specimens examined

Institutional Abbreviations used:CAS, California Academy of Sciences, San Francisco, USA; DE, Faculty of Natural Sciences, Comenius University in Bratislava, Slovakia; MNHN, Muséum national d'Histoire naturelle, Paris, France; NHMW, the Natural History Museum Vienna (Austria); PEM, Port Elizabeth Museum, Port Elizabeth, South Africa; R, the Bayworld Porthe Elisabeth collection in South Africa; RMCA, Royal Museum for Central Africa, Tervuren, Belgium; SMF, Senckenberg Natural History Museum, Frankfurt, Germany; UF, University of Florida, Gainesville, USA;UMMZ, University of Michigan, Ann Arbor, USA; USNM, National Museum of Natural History, Smithsonian Institution, Washington, DC, USA; UTEP, The University of Texas at El Paso, El Paso, USA; ZFMK, The Zoological Research Museum Alexander Koenig, Bonn, Germany.

The following specimens of extant agamids (outgroup): and chameleonids have been used for comparison (Supplementary Figs. 14-16): *Uromastyx geyri* (UF 44229); *Uromastyx* sp. (A.Č. pers.coll.); *Archaius tigris* (MNHN 1989.2872 adult female); *Bradypodion thamnobates* (NHMW 39729); *Bradypodion occidentale* (MNHN 1998.379 & 2000.2530 Adults); *Bradypodion setaroi* (R6921); *Brookesia perarmata* (MNHN 1993.0165 adult female); *Brookesia superciliaris* NHMW 7380); *Calumma globifer* (DE 82 Adult); *Ca. ambreense* (ZFMK 53845), *Ca. parsonii* (UF 76848), *Calumma nasutum* (MNHN 6643F Adult); *Calumma brevicorne* (USNM 163511); *Calumma boettgeri* (CAS 156894); *Chamaeleo namaquensis* (MNHN 282); *Chamaeleo africanus* (UMMZ 014181 Adult); *Chamaeleo chamaeleon* (DE 66), *Chamaeleo calyptratus* (DE 65, DE 74, DE 75, DE 76, DE 77); *Chamaeleo dilepis* (MNHN 2005.3341); *Chamaeleo zeylanicus* (CAS 232061); *Furcifer campani* (ZFMK 61143); *Furcifer pardalis* (DE 80 – 81); *Furcifer oustaleti* (USNM 149252; SMF 73684; SMF 59447; SMF 73685 and A.H. pers. coll.); *Kinyongia xenorhina* (UTEP 547; RMCA R-85003.0014); *Kinyongia fischeri* (C.V.A. pers.coll.); *Nadzikambia mlanjensis*

(PEM 16294 & PEM 18445 Adult male and female); *Palleon nasus* (MNHN-RA-1971.274); *Rhampholeon spectrum* (NHMW 10196); *Rhampoleon boulengeri* (RMCA R-26570 Adult male); *Rieppeleon brevicaudatus* (UF 65355); *Rieppeleon kerstenii* (NHMW 7376); *Trioceros cristatus* (RMCA R-28176 Adult); *Trioceros jacksonii* (SMF 90037; A.Č. pers.coll. 2 Adult males and one female); and *Trioceros johnstoni* (RMCA R-14150 Adult male).

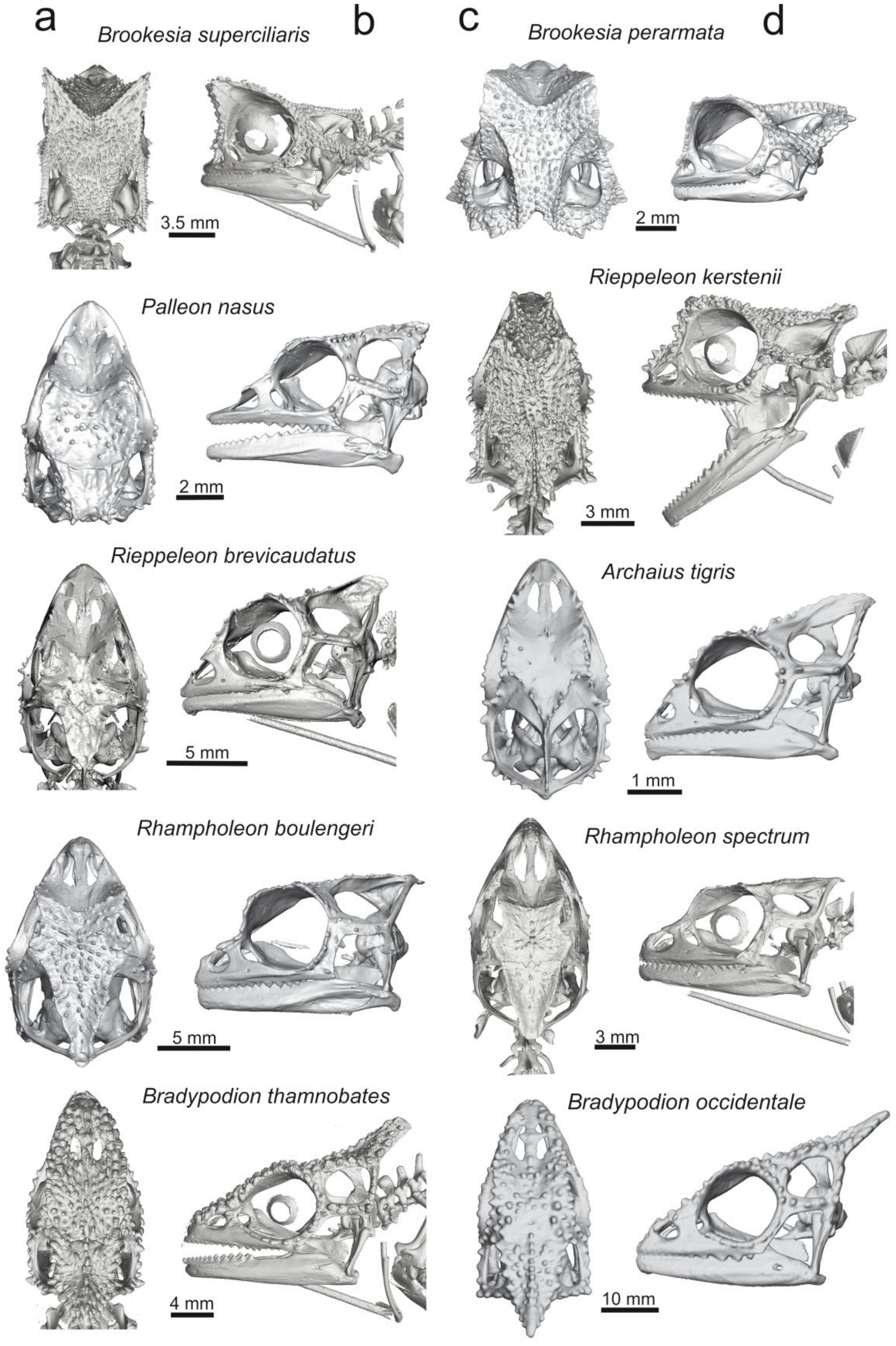

Supplementary Fig. 14 Skulls of selected species of extant chamaeleonid genera *Brookesia*, *Palleon*, *Rieppeleon*, *Archaius*, *Rhampholeon* and *Bradypodion* in dorsal (**a** and **c** columns) and lateral (**b** and **d** columns) views.

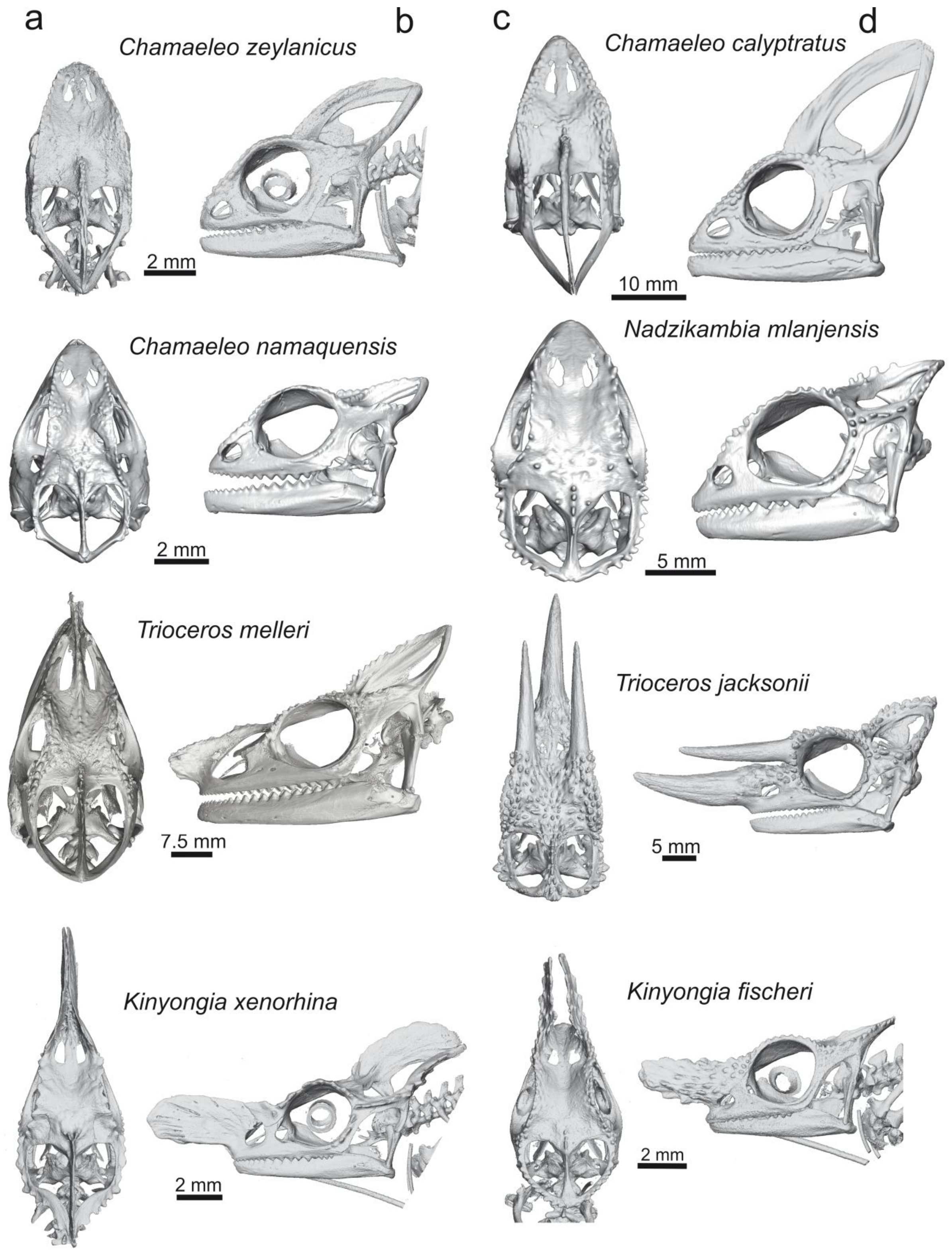

Supplementary Fig. 15 Skulls of selected species of extant chamaeleonid genera *Chamaeleo*, *Nadzikambia*, *Trioceros* and *Kinyongia* in dorsal (**a** and **c** columns) and lateral (**b** and **d** columns) views.

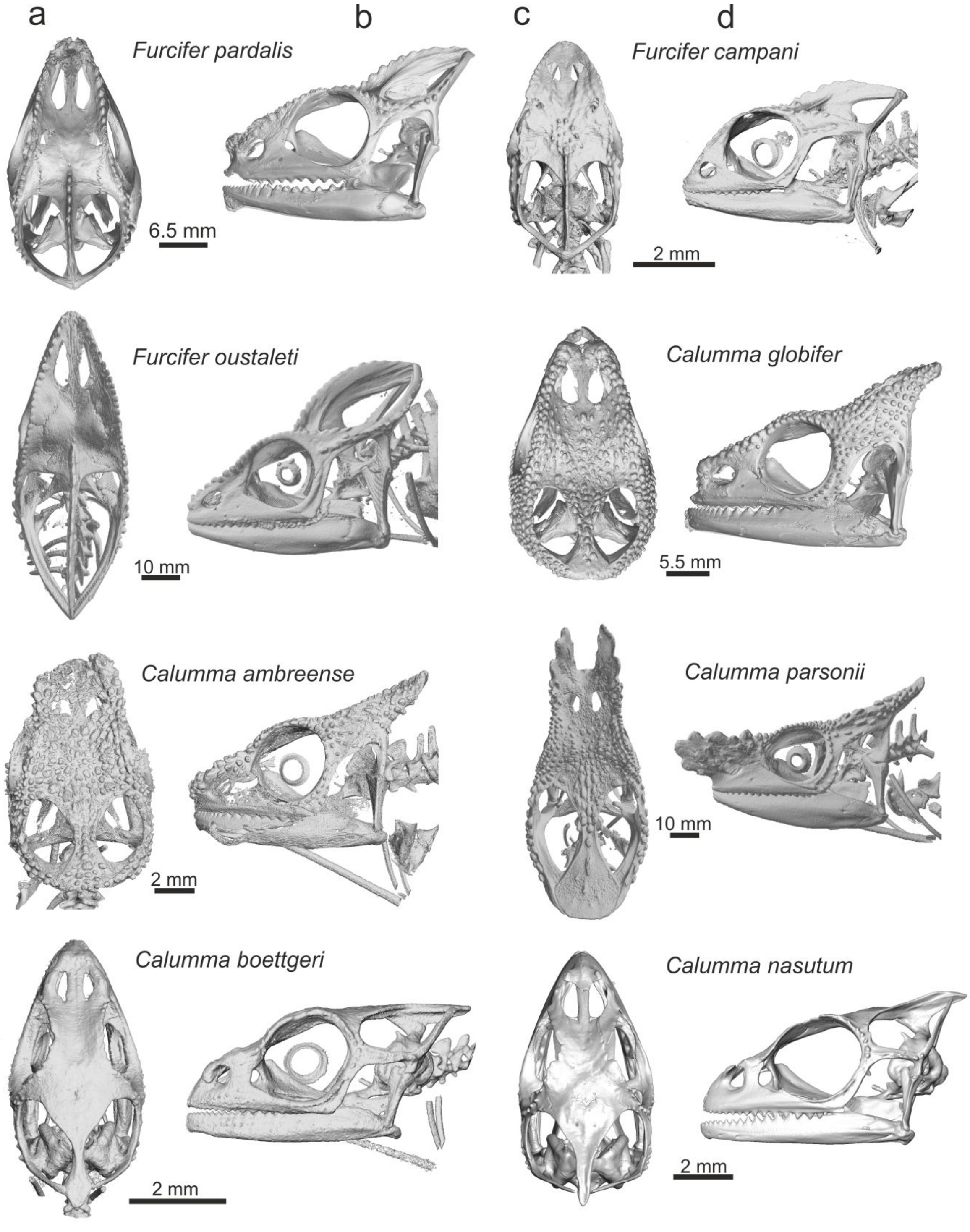

Supplementary Fig. 16 Skulls of selected species of extant chamaeleonid genera *Furcifer* and *Calumma* in dorsal (**a** and **c** columns) and lateral (**b** and **d** columns) views.

4. Data Matrix

The list of characters comes from Rieppel and Crumly[22] + newly added characters:

1. Nasals paired (0) or fused (1).

2. Premaxilla separate (0) or in contact with frontal (1).

3. Nasal enters external naris (0) or is separated from it by a prefrontal fontanelle (l), whose lateral rim is formed by the contact of the prefrontal and the maxilla, 0s the external naris is confluent with the fontanelle (2).

4. The prefrontal and postorbitofrontal remain separated along the dorsal margin of the orbit in the outgroups with a T-shaped frontal (0).

5. *Descensus lateralis parietalis* and *crista parietalis inferior* are flanges of bone that extend inferiorly from the dorsal plate of the parietal (see Rieppel, 1987, for a detailed description). The *descensus lateralis parietalis* are paired, whereas the *crista parietalis inferior* is a single median plate. In the outgroups, only the *descensus lateralis parietalis* is present (state 0). Both flanges are fused and present (state 1) in some *Rhampholeon*. They are separate and present (state 2) only in *Brookesia.* In the majority of chameleons the *descensus lateralisparietalis* is reduced or absent, leaving only the *crista parietalis inferior* (state *3).*

6. Parietal contacts prootic and supraoccipital (0) or the supraoccipital only (1).

7. Parietal may (1) or may not (0) form a posteriorly narrowing terminus.

8. Parietal with (0) or without (1) supratemporal processes.

Brookesia are the only chamaeleonines that have supratemporal processes, which extend posteroventrally from the rear edge of the parietal to meet the squamosal.

9. Dorsal process of the squamosal does (0) or does not (1) meet the parietal.

10. Supratemporal present (0) or absent (1).

11. Squamosal does (0) or does not (1) meet the jugal.

12. Lacrimal absent (1) or present (0).

13. Maxillae do (1) or do not (0) meet on the dorsal midline of snout.

14. Maxillae do (1) or do not (0) meet on ventral midline of palate.

15. Vomer paired (0) or partly or wholly fused (1).

16. Surangular separate (0) or fused (1) to prearticular.

represented by a single element.

17. Angular present (0) or absent (1).

18. Tooth row restricted to level anterior to coronoid process (0) or extending posteriorly beyond the

anterior margin of the coronoid process (1).

19. Maxilla does (1) or does not (0) separate premaxilla from nasal.

20. Prefrontal fontanelle absent *(0);* prefrontal fontanelle present and frontal does (2) or does not (I)

enter the prefrontal fontanelle or the posteriorly expanded external naris, respectively.

21. Lung aseptate (0), with small septae (l), with large septae (2).

22. Calyces on hemipenis present (0) or absent (1).

23. Apical ornamentation of hemipenis absent (0), dual (l), quadruple (2).

24. Apical ornamentation absent (0), by crests (l), horns (2), pedunculi and auriculae (3), or otherwise (4).

New Added Characters

25. Synapophyses on the axis[23]: absent (0), present (1).

26. Hourglass-shaped parietal (mid-lateral constriction) absent (0), present (1).

27. Maximum width of frontal in posterior region: with well separated posterolateral processes (0), in posterior region, but posterolateral processes are not well separated (1), anteriorly to the posterior region (2).

28. The posterodorsal process of postorbitofrontal reaches above the orbit and upper temporal fenestra is not visible in lateral view (0), reaches the upper part of the orbit, fenestra only partially visible (1), reaches around mid-region of the orbit, fenestra well-visible (2).

29. The parietal forms a dorsally elevated casque: absent (0), present small (1), large (2).

30. Prefrontal fenestra absent (0), present but separated from external naris (1), present and connected to external naris.

31. Ornamentation on parietal (0) weak or absent; well developed, broadly arranged (1), forming a single crest (2), forming a letter Y (3), forming a letter psi (4).

32. Central region of frontal well ornamented by protuberances absent (0), present (1).

33. Prefrontal in dorsal view: laterally inclined (0), anteriorly or medially oriented (1).

34. Orbital margin in dorsal view markedly rounded (0), more or less straight (1).

35. Facial process of maxilla dorsally oriented (0), anteriorly inclined (1).

36. Suborbital process of jugal in lateral aspect largely exposed (0), only a dorsal thin portion exposed (1), covered by maxilla (2).

37. Narrow skull (its width is less than 50% of the max length) absent (0), present (1).

38. The maxilla protrudes anteriorly into protuberances or horn: absent (0), present (1).

49. Skull in dorsal view rather ovoid shape (0), rectangular shape (1).

40. Extremely long posterodorsal process of the atlas[23]: absent (0) present (1).

41. Markedly anteroventrally inclined dorsal margin of the neural spine of the axis[23]: absent (0), present (1).

42. Prefrontal protrudes anteriorly into a horn absent (0) present (1).

43. Posterodorsal process of postzygapophyses[23]: absent (0) present (1).

5. Geometric morphometric analyses

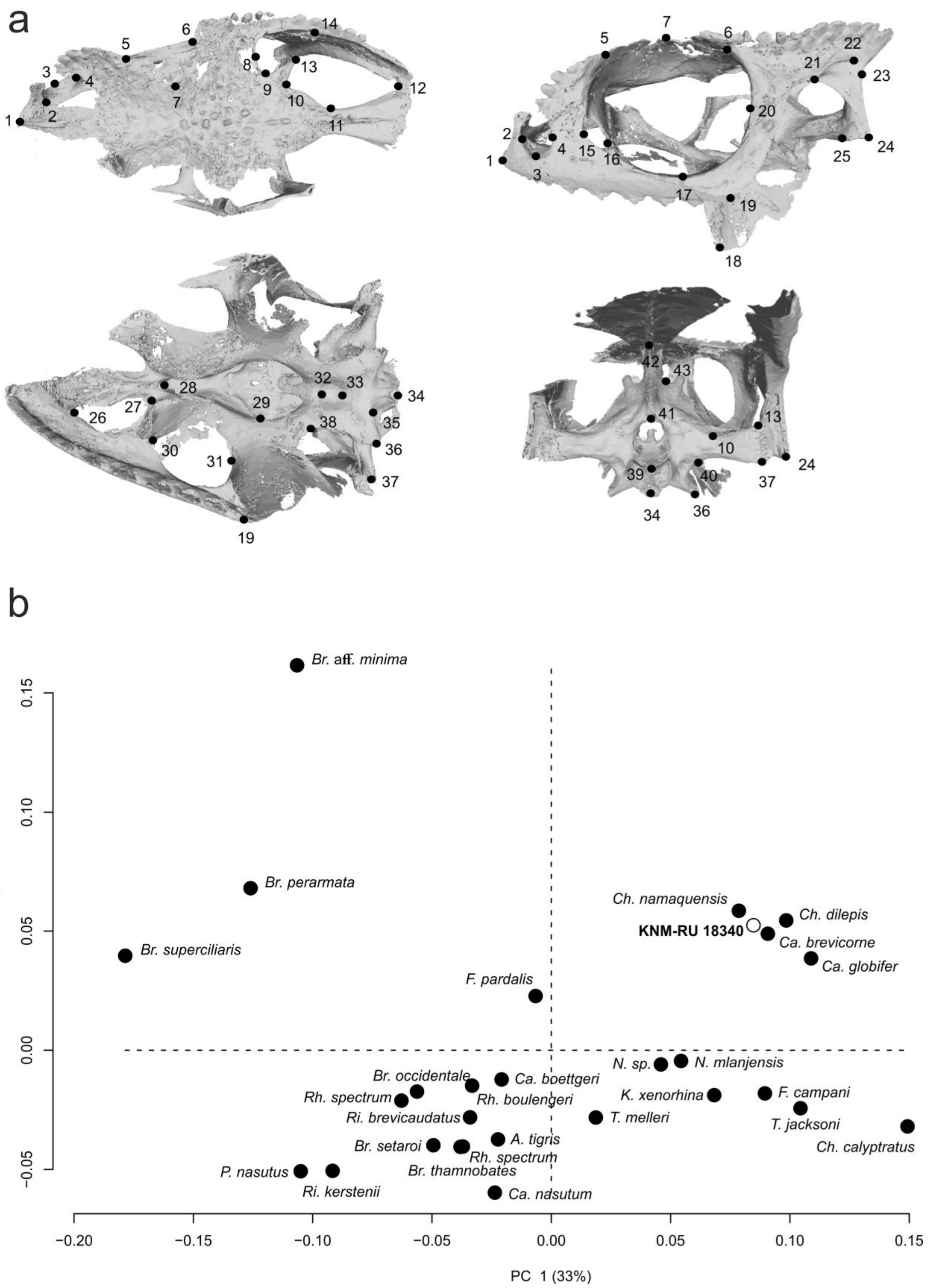

Supplementary Fig. 17 a Figure illustrating the landmarks taken on the skull of the fossil. As the fossil was partially damaged, only landmarks visible on the fossil could be take on the

skulls of the extant species. Landmarks were taken to characterize the overall shape variation in the skull. See supplementary table 1 for description of the landmarks. **b** Results of the principal components analysis performed on the 3D geometric morphometric data after a generalised Procrustes superimposition. Illustrated are the first two PC axes, jointly explaining 44% of the variance in the data. The fossil is illustrated by the open symbol and clusters close to a group of species including *Calumma brevicorne*, *Calumma globifer*, *Chamaeleo dilepis* and *Chamaeleo namaquensis*.

Supplementary Tab. 1 Landmark definitions; all landmarks were taken on the right side of the specimen as that side was the most intact in the fossil.

**Landmark number**	**description**
1	Anteriormost point of the premaxilla.
2	Anteriormost point of the external naris.
3	Ventralmost point of the external naris.
4	Posteriormost point of the external naris.
5	Anterodosral point of the orbit.
6	Posterodorsal point of the orbit.
7	Dorsalmost point of the orbit (contact between postorbitofrontal and prefrontal).
8	Anteriormost point of the adductor chamber.
9	Anterodorsal point of the proximate part of the otooccipital.
10	Posterodorsal point of the proximate part of the otooccipital.
11	Medialmost point of the adductor chamber on the parietal.
12	Posteriormost point of the adductor chamberat the contact between squamodal and parietal.
13	Posterodorsal point of the distal part of the paraoccipital process.
14	Lateralmost point of the adductor chamber at the contact between squamosal and postorbitofrontal.
15	Anteriormost point of the orbit on the maxilla.
16	Point on the ventral part of the palatine process of the premaxilla across from point 15.
17	Ventralmost point of the orbit.
18	Ventralmost point of the pterygoid wing.
19	Posteriormost point of the maxilla.

20	Posteriormost point of the orbit.
21	Maximum of curvature at the postorbitofrontal-squamosal junction.
22	Maximum of curvature at the intersection of the three processes of the squamosum.
23	Posterodorsal part of the ventral process of the of the squamosal.
24	Distalmost part of the ventral process of the squamosal.
25	Anteriormost point of the supratemporal.
26	Lateralmost point of the vomer.
27	Anteriormost point of the maxillary process of the palatine across the constriction from point 26.
28	Midline point at the suture between left and right palatines.
29	Maximum of curvature on the pterygoid.
30	Point at the ventralmost aspect of the palatine at the anterior part of the suborbital fenestra.
31	Posteriormost point of the suborbital fenestra on the ectopterygoid.
32	Posterior point of the parasphenoid.
33	Anterior point of the sphenoid.
34	Posteriormost point of the basioccipital.
35	Maximum of curvature on the basioccipital.
36	Lateralmost point of the basioccipital (basal tubercle).
37	Posteroventral point of the distal part of the paraoccipital process.
38	Maximum of curvature on the pterygoid.
39	Ventralmost point of the occipital foramen.
40	Maximum of curvature on the otooccipital.
41	Dorsalmost point of the occipital foramen.
42	Dorsalmost point of the ascending process of the supraoccipital.
43	Anteriormost point of the supraoccipital; maximum of curvature medial to the anterolateral process.

6. Character Reconstruction

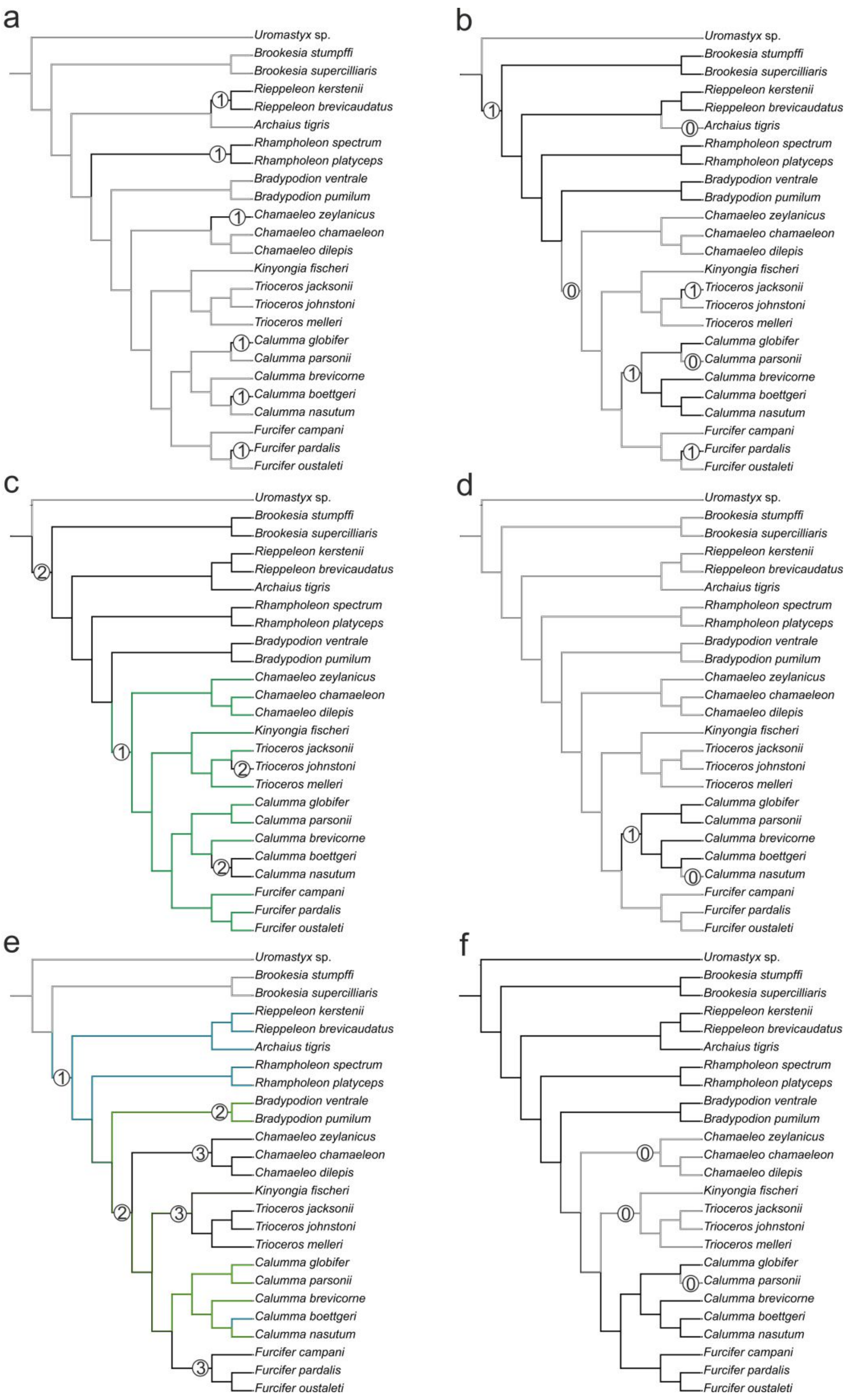

Supplementary Fig. 18 Trace character history analysis in Mesquite: **a** the premaxilla separate (0) or in contact with frontal (1); **b** squamosal does (0) or does not meet jugal (1); **c** the prefrontal and postorbitofrontal separated, T shaped frontal (0) or prefrontal does (1) or does not meet postorbitofrontal (2); **d** the hour-glass shaped parietal (mid-lateral constriction)

absent (0), present (1); **e** the parietals forms (0) posteriorly narrowing terminus; or not (1), dorsoventrally compressed (2); laterally compressed (3); **f** the lacrimal absent (1) or present (0).

7. References

1. Shackleton, R. M. A contribution to the geology of the Kavirondo Rift valley. *Q. J. Geol. Soc. Lond.* **16**, 345–392 (1951).
2. McCall, G. J. H. Geology of the Gwasi area. Geological Survey of Kenya, Report 45 (1958).
3. Van Couvering, J. A. Geology of Rusinga Island and Correlation of the Kenya Mid-Tertiary Fauna (Ph.D. Dissertation, Cambridge University, 1972).
4. Le Bas, M. J. & Rubie, D. C. Kisingiri II: The sub-volcanic alkali silicate rocks. (ed LeBas, M. J.) Carbonatite-Nephelinite Volcanism (Wiley, New York, pp. 47–97, 1977).
5. Pickford, M. Cainozoic paleontological sites of western Kenya. *Münch. Geowiss. Abh. A, Geol. Paläontol.* **8**, 1–151 (1986).
6. Bestland, E. A., Thackray, G. D. & Retallack, G. J. Cycles of Doming and Eruption ofthe Miocene Kisingiri Volcano, Southwest Kenya. *J. Geol.* **103**, 598–607 (1995).
7. Peppe, D. J., McNulty, K. P., Cote, S. M., Harcourt-Smith,W. E. H., Dunsworth, H. M. & Van Couvering, J. A. Stratigraphic interpretation of the Kulu Formation (Early Miocene, Rusinga Island, Kenya) and its implications for primate evolution. *J. Hum. Evol.* **56**, 447–461 (2009).
8. Rieppel, O., Walker, A. & Odhiambo, I. A preliminary report on a fossil Chamaeleonine (Reptilia: Chamaeleoninae) skull from the Miocene of Kenya. *J. Herpetol.* **26**, 77–80 (1992).

9. Drake, R. E., Van Couvering, J. A., Pickford, M. H., Curtis,G. H. & Harris, J. A. New chronology for the Early Miocene mammalian faunas of Kisingiri, Western Kenya. *J. Geol. Soc.***145**, 479–491 (1988).

10. Peppe, D. J., Deino, A. L., McNulty, K. P., Lehmann, T., Harcourt-Smith, W. E. H., Dunsworth, H. M. & Fox, D. L. New age constraints on the early Miocene faunas from Rusinga and Mfangano Islands (LakeVictoria, Kenya). *American Association of Physical Anthropologists*. Abstract (2011).

11. Peppe, D. J., Deino, A. L., McNulty, K. P., McCollum, M. S., Mitchell, A. L., Driese, S. G., Dunsworth, H. M., Fox, D. L., Harcourt-Smith, W. E., Jenkins, K., Lehmann, T. & Michel, L. A. Revised geochronology of the Early Miocene faunas from Rusinga Island and Mfangano Island (Lake Victoria, Kenya): implications for Miocene hominoid evolution and faunal succession. *Am. J. Phys. Anthropol.* **162**, 313 (2017).

12. McCollum, M. S., Peppe, D. J., McNulty, K. P., Dunsworth, H. M., Harcourt-Smith, W. E. H. & Andrews, A. L. Magnetostratigraphy of the early Miocene Hiwegi Formation (Rusinga Island, Lake Victoria, Kenya). *Geol. Soc. Am., Abstracts with Programs* **44**, 241 (2012).

13. Pickford, M. Preliminary Miocene mammalian biostratigraphy for Western Kenya. *J. Hum. Evol.* 10, 73–97 (1981).

14. Pickford, M. Kenya Palaeontology Gazetteer, vol. 1: Western Kenya. Kenya National Museums Spec. Publ. 1. (National Museums of Kenya, Department of Sites and Monuments Documentation, Nairobi, 1984).

15. Andrews, P., Begun, D. & Zylstra, M. Interrelationships between functional morphology and paleoenvironments in Miocene hominoids. (eds Begun, D.,Ward, C. & Rose, M.) Function, Phylogeny, and Fossils: Miocene Hominoid Evolution and Adaptations (Plenum Press, New York, pp. 29–58. 1997).

16. Maxbauer, D. P., Peppe, D. J., Bamford, M., McNulty, K. P., Harcourt-Smith, W. E. H. & Davis, L. E. A morphotype catalog and paleoenvironmental interpretations of early Miocene fossil leaves from the Hiwegi Formation, Rusinga Island, Lake Victoria, Kenya. *Palaeontol. Electron.* **16**, 28A (2013).

17. Michel, L. A., Peppe, D. J., Lutz, J. A., Driese, S. G., Dunsworth, H. M., Harcourt-Smith, W. E. H., Horner, W., Lehmann, T., Nightengale, S., McNulty, K. P. Remnants of an ancient forest provide ecological context for Early Miocene fossil apes. *Nature Comm.* **5**, 3236 (2014).

18. Peppe, D. J., McNulty, K. P., Deino, A. L., Michel, L. A., McCollum, M. S., Driese, S. G., Dunsworth, H. M., Harcourt-Smith, W. E, Jenkins, K. E. & Lehmann, T. Early Miocene paleoenvironments of the Hiwegi Formation on Rusinga Island (equatorial Africa, Lake Victoria, Kenya) and their implications for hominoid evolution: *Geol. Soc. Am. Abstracts with Programs* 48(7): doi: 10.1130/abs/2016AM-280981 (2016).

19. Garrett, N. D., Fox, D. L., Peppe, D. J., Michel, L. A, Lehmann, T. & McNulty, K. P. Stable Isotope Ecology of the Early Miocene Rusinga Island Mammalian Communities from the Kulu and Hiwegi Formations. (submitted).

20. Michel, L. A., Lehmann, T., McNulty, K. P., DiPietro, L., Driese, S. G., Dunsworth, H., Fox, D. L., Harcourt-Smith, W. E., Jenkins, K. & Peppe, D. J. Sedimentological and paleoenvironmental study from Waregi Hill in the early Miocene Hiwegi Formation, Lake Victoria, Kenya and the influence on early ape evolution. (submitted).

21. Čerňanský, A., Smith, K. T. & Klembara, J. Variation in the position of the jugal medial ridge among lizards (Reptilia: Squamata): its functional and taxonomic significance. Anat Rec. **297**, 2262–2272 (2014).

22. Rieppel, O. & Crumly, C. Paedomorphosis and skull structure in Malagasy chamaeleons (Reptilia: Chamaeleoninae). *J. Zool.* **243**, 351–380 (1997).

23. Čerňanský, A., Boistel, R., Fernandez, V., Tafforeau, P., Le Noir, N. & Herrel, A. The atlas-axis complex in chamaeleonids (Squamata: Chamaeleonidae), with description of a new anatomical structure of the skull. *Anat. Rec.* **297**, 369–396 (2014).
